# Membrane filtration reduces nutrient availability and invasion potential in drinking water systems, without affecting mature biofilms

**DOI:** 10.3389/fmicb.2025.1622038

**Published:** 2025-08-13

**Authors:** Thomas Pluym, Fien Waegenaar, Karlien Dejaeger, Marie Dhoore, Eline Mestdagh, Emile Cornelissen, Nico Boon, Bart De Gusseme

**Affiliations:** ^1^Center for Microbial Ecology and Technology (CMET), Department of Biotechnology, Ghent University, Ghent, Belgium; ^2^Center for Advanced Process Technology for Urban Resource Recovery (CAPTURE), Ghent, Belgium; ^3^The Particle and Interfacial Technology Group (PaInT), Department of Green Chemistry and Technology, Ghent University, Ghent, Belgium; ^4^KWR Water Research Institute, Nieuwegein, Netherlands; ^5^Farys, Department Innovation Water – R&D, Ghent, Belgium

**Keywords:** drinking water, biostability, microbiology and biofilm, membrane filtration, nutrient limitation

## Abstract

Ensuring biostable drinking water is a growing priority for drinking water utilities, especially in non- or minimally chlorinated distribution systems where microbial regrowth is controlled through nutrient limitation. In this study, we evaluated the efficacy of ultrafiltration (UF) and nanofiltration (NF) in reducing total organic carbon (TOC) and their impact on the microbiology in a pilot-scale drinking water distribution system over 7 weeks. NF achieved significantly higher TOC removal (75.4%) compared to UF (25.4%), with high performance size exclusion chromatography revealing almost complete removal of all molecular weight fractions in NF-treated water. When introduced into the pilot system, NF-, UF-treated water, and untreated tap water supported similar increasing bulk cell concentrations, but exhibited distinct bacterial community compositions, with NF-treated water showing the most divergent microbiome. Despite these differences in the bulk water, the mature biofilm community (~2 years old) remained stable, underscoring it resilience to changes in nutrient conditions. An invasion assay demonstrated that decay rates of unwanted microorganisms increased with decreasing organic carbon content. For example, decay rates for the introduced microorganism *Pseudomonas putida* in NF-, UF- treated water, and untreated tap water were respectively, −0.18 h^−1^, −0.143 h^−1^, and −0.089 h^−1^, indicating enhanced biostability in membrane-treated systems.

## 1 Introduction

Drinking water has undergone an extensive production process before it reaches the consumer, and even more importantly it has travelled through an immense and complex drinking water distribution network, consisting of a high-pressure network, water towers, pumping stations, and low-pressure pipelines. Receiving safe and qualitative drinking water from the tap, is thus not evident, even if access to safe drinking water was declared a basic human right by the United Nations (World Health Organization, 2022). Consequently, in recent decades, drinking water providers have strived to ensure safe and qualitative water at the tap. A current approach to reach this is the production and distribution of biologically stable drinking water, ensuring that the water reaching the customer maintains a microbiological quality comparable to the quality directly after production with an acceptable degree of change. This stability involves establishing a microbial community capable of resisting environmental changes and unexpected events within drinking water distribution systems (DWDS), with the ultimate goal of preventing disease transmission (Favere et al., 2021; Prest et al., 2016). This means that during production the drinking water providers do not only focus on the removal of micro-organisms that could be harmful to the customer, but try to remove substances that could cause microbial (re)growth in DWDS that compromise water quality (Prest et al., 2016; Overheid, 2023).

One approach to ensuring biostable water in DWDS is chlorine disinfection with a high residual chlorine concentration of up to 5 mg/L Cl₂, local regulations (depends on the country), the quality of the source water, the treatment processes, and other relevant factors (World Health Organization, 2022; Zhang et al., 2002). In Europe, free chlorine concentrations are regulated to be lower. More specifically, in Flanders (Belgium), the amount of chlorine disinfectant is based on a legal upper limit of 0.25 mg/L residual chlorine at the customer’s tap (Krasner et al., 2016; VMM, 2022). However, the use of chemical disinfection could lead to the production of mutagenic and carcinogenic disinfection byproducts (DBPs) formed by the reaction of the disinfectant with organic matter compounds, ultimately deteriorating the overall water quality (Li and Mitch, 2018; Richardson and Postigo, 2012; Song et al., 2021; Zhang and Minear, 2006). Furthermore, when the disinfectant residual is depleted, the nutrients released from oxidized organic matter and decomposed biomass foster microbial regrowth, increasing risk of drinking water quality deterioration and, consequently, the risk of disease (Bertelli et al., 2018; Boon et al., 2023; Chatzigiannidou et al., 2018).

Today, one of the key approaches to produce biologically stable water in non- or minimally chlorinated distribution systems (e.g., <0.25 mg/L in Flanders) involves extensive nutrient limitation. To achieve this, drinking water providers primarily reduce the levels of available organic compounds (e.g., organic carbon, humic substances) and, to a lesser extent, inorganic nutrients such as phosphorus, nitrogen, and trace elements (Ihssen and Egli, 2004; Prest et al., 2016; Schmidt et al., 1998). These nutrients are essential for the survival and multiplication of heterotrophic bacteria in the bulk water phase, but also for the colonization of the drinking water distribution system (Polanska et al., 2005). Since the presence of natural organic matter (NOM), and more specifically, assimilable organic carbon (AOC) is found to be a crucial parameter for microbial (re)growth and biological stability in drinking water, nutrient removal and limitation is particularly important (Hammes et al., 2009; Park et al., 2016; Polanska et al., 2005; Schurer et al., 2022). These findings underline the overall importance of nutrient limitation to ensure high drinking water quality.

Traditionally, drinking water treatment heavily relies on biological filtration systems to remove those nutrients and pollutants, such as slow sand filtration, which became popular because of their ease of use, overall performance and energy effectiveness (Guchi, 2015). Nowadays, drinking water providers start to use one or multiple membrane filtration techniques in their treatment train, such as ultrafiltration (UF) (pore size: 0.02–0.05 μm), nanofiltration (NF) (pore size: 0.001–0.01 μm), and reverse osmosis (RO) (pore size: ±0.0005 μm), particularly chosen because of their high permeate quality (Gao et al., 2011; Ma et al., 2023; Van Der Bruggen et al., 2003). While UF is mostly used to NOM, NF is important for the removal of divalent ions, such as Ca^2+^ and heavy metals (He et al., 2019). By reducing the high molecular-weight (MW) carbon compounds and, consequently, the regrowth potential (biomass production potential and bacterial growth potential), UF as a post-treatment step to a conventional treatment could be a promising technique to ensure biological stability (Schurer et al., 2019). Not all organic material can be used for growth of bacteria, as generally, it is mostly the low MW fraction that can be degraded by bacteria (Van Der Kooij and Hijnen, 1984). However, in a previous study on a large-scale distribution network, a positive correlation between particulate high MW carbon and microbial regrowth was found (Hijnen et al., 2018). To remove these compounds and other micropollutants, membrane filtration techniques can be used, reducing the amount of available nutrients and the potential for microbial regrowth. Additionally, membrane treatment has the advantage of having a small footprint and automation, and it is adaptable to various feed streams and allows for a wide-spectrum removal of pollutants. However, a high operational cost, membrane fouling, and long-term stability of the membranes have to be considered (Guo et al., 2022).

A previous full-scale study showed that introducing UF treated water into a DWDS that had previously been supplied with conventionally treated water, triggered microbial release from the biofilm. Prior to this change, the microbial community was composed of bacteria originating from the treatment plant, while the change, the community was dominated by DWDS-associated microbes (Chan et al., 2019). Lab scale experiments and experiments on model organisms such as *Pseudomonas aeruginosa* and *Pseudomonas putida* have demonstrated that, when carbon sources are depleted, biofilm formation is limited and so-called dispersal may be initiated, resulting in a release of bacterial cells from the biofilm (De Plano et al., 2024; Díaz-Salazar et al., 2017; Nair et al., 2021). Although multiple studies have focused on biofilm formation and adhesion of model organisms under high nutrient conditions and under low nutrient conditions, apart from specific case-studies, there is limited knowledge about the effect of nutrient limitation on an existing mature, and more importantly complex and diverse drinking water biofilm (Chan et al., 2019; Hu et al., 2025). With the emerging properties of mature biofilms in mind (i.e., stable against environmental stressors), we hypothesize that distributing UF- or NF-treated water through DWDS with a mature biofilm (i.e., stable cell density and community composition) may alter bulk microbiology and bacterial survival within the system while leaving the robust biofilm largely unchanged (Boe-Hansen et al., 2002b, 2002a; Waegenaar et al., 2024b). This hypothesis is based on previous experiments examining various flushing regimes, biofilm growth at different temperatures, and the conversion of odor and taste precursors, which showed that mature biofilms are resilient (Mol et al., 2025; Waegenaar et al., 2025, 2024b).

The primary objective of this study was to assess how nutrient depletion, more specifically organic carbon limitation, impacts potential regrowth and the ability of unwanted microorganisms to invade and persist in DWDS. To do so, UF- and NF-treated water was introduced into a pilot-scale, low-pressure distribution network with a well-characterized mature biofilm (proven to be stable in cell density and community composition after 100 days, as described in Waegenaar et al. (2024b)) and biofilm and bulk water microbial community was followed.

## 2 Materials and methods

### 2.1 Experimental design

Over a period of 7 weeks (from 14-03-2024 till 02-05-2024), a DWDS pilot with a mature biofilm (consistent cell concentrations and equilibrium between growth, attachment and detachment (Boe-Hansen et al., 2002b; Waegenaar et al., 2024b)) was supplied with UF-treated water, NF-treated water, and as a control, untreated tap water. The water used for the experiments was chlorinated tap water from Farys (Ghent, Belgium), sourced from the Albert Channel (surface water) in Antwerp (Belgium). By the time the water reached Ghent, where the pilot facility is located, the free chlorine concentration was below the detection limit. The distribution pilot consisted of three parallel and identical subsystems (loops). Each loop was connected to a 1 m^3^ non-transparent high density polyethylene intermediate bulk container (IBC), which was not disinfected beforehand, connected to 100 m of unplasticized polyvinylchloride pipes with a diameter of 80 mm (García-Timermans et al., 2023) (Supplementary Figure S1). Water from the IBC was pumped into the loops, recirculated through the system, and subsequently returned to the IBC. Each week, NF- and UF-treated tap water was produced using a fractionation membrane unit (described in Section 2.1.1). Loop 1 was fed with NF-treated tap water, loop 2 with UF-treated tap water, and loop 3 with untreated tap water (750 L). The water recirculated through the system for 7 days, corresponding to the hydraulic residence time. After this period, the water was discharged, and fresh treated or untreated water was added to the system. After 6 weeks of operation, an invasion experiment was conducted, introducing unwanted microorganisms into the system. Throughout the experiment, the flow velocity was maintained at 0.08 m/s, the temperature at 16°C, and the pressure was kept between 0.7 and 0.9 bar, in line with previous studies (Supplementary Figures S2, S3) (Husband et al., 2008).

**Figure 1:**
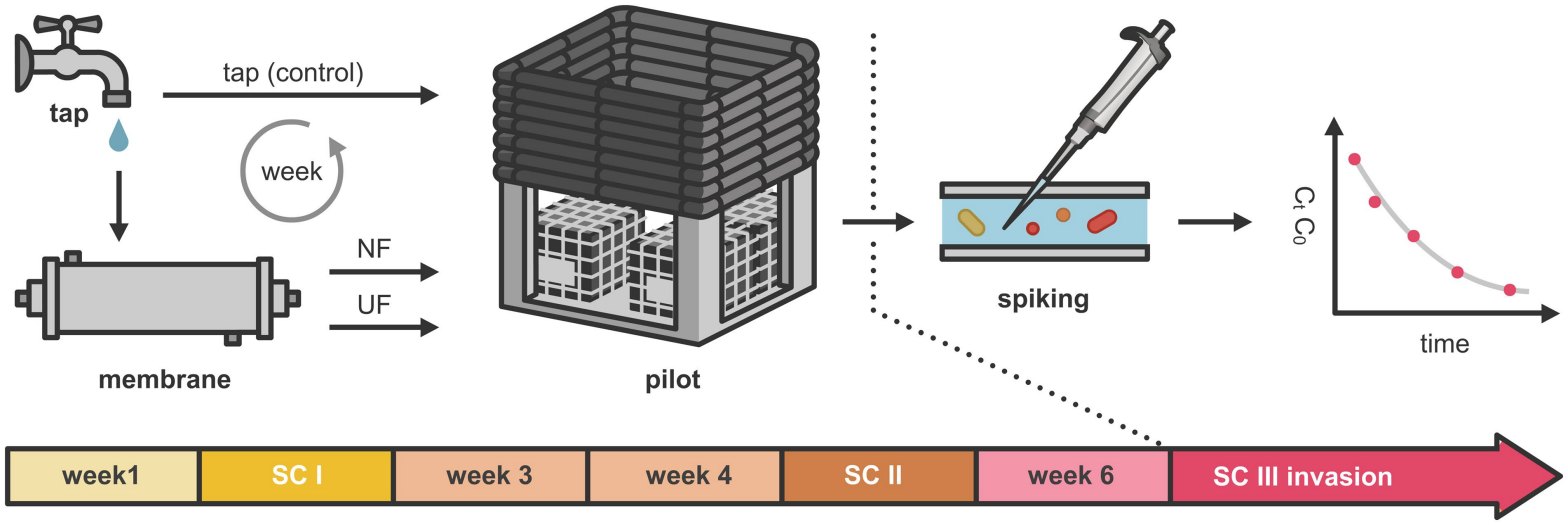
Overview of the experimental setup. Over a period of 7 weeks, a DWDS pilot was supplied with NF-treated, UF-treated, and untreated tap water. Three sampling campaigns were performed, referred to as sampling campaign I (SC I), sampling campaign II (SC II), and sampling campaign III (SC III), corresponding to weeks 2, 5, and 7, respectively. In sampling campaign III, selected unwanted microorganisms were introduced into the system.

Three sampling campaigns, referred to as sampling campaign I (SC I), sampling campaign II (SC II), and sampling campaign III (SC III), were performed over the course of 7 weeks (Figure 1). These were carried out in week two (after 1 week of conditioning), in week five, and in week seven (during the invasion week). In all of those weeks, samples were collected after 1 h, 1, 4, and 7 days after the addition of treated and fresh tap water. During sampling campaign III, besides sample collection, an invasion experiment was carried out, in which unwanted microorganisms were spiked. The samples taken during all sampling campaigns were analyzed for TOC, HPSEC-TOC, and IC. Additionally, microbial dynamics of the bulk water were followed using online flow cytometry. Samples for 16S rRNA gene-based amplicon sequencing were taken 1 h and 7 days after the introduction of (treated) water. In addition, mature biofilm samples (*n* = 2) (coupons that were already in the pilot before) were taken before the start of the experiment (day 0), after 21 and after 42 days. Besides that, young biofilm samples (grown over the course of the experiment) were taken at day 42 (before the invasion) and day 49 (after the invasion experiment). They were analyzed using flow cytometry and 16S rRNA gene-based amplicon sequencing. Prior to this experiment, several experiments were conducted on the pilot installation over a 1.5 year. These experiments demonstrated the system’s stability, as indicated by a consistent biofilm community and stable bulk water cell concentrations (García-Timermans et al., 2023; Mol et al., 2025; Waegenaar et al., 2024b, 2025).

#### 2.1.1 Fractionation membrane unit

Crossflow UF and NF was performed on a pilot-scale unit with a 2,540-type spiral wound membrane element (2.5″) with an active filtration area of 2.7 m^2^ (Supplementary Figure S4). Feed and permeate tubing were connected to 1 m^3^ IBCs to ensure sufficient volume production. Each week, 1,000 L of tap water was treated using UF and NF membranes, achieving each a recovery rate of 75%. To do so, the concentrate stream was sent back to the feed IBC for recirculation and the permeate was collected in the IBC as feed water of the pilot-scale DWDS. The UF membrane was a Synder ST-2540HM membrane (polyether sulfone, molecular weight cutoff (MWCO) 10,000 Da; Synder, California, USA), and UF was conducted at a feed flow rate of 5.7 L/min at 4 bar. The NF membrane was a Trisep XN45 membrane (polypiperazine amide, MWCO 300–500 Da; Trisep, California, USA), and NF was done at a feed flow rate of 5.7 L/min at 7.5 bar. The membrane setup was equipped with Grafana-based logging system, enabling the continuous logging of parameters such as permeate flux, mass transfer coefficient, pressure, and conductivity.

When the water treatment setup was not in use, the membrane remained in the system, where it was preserved in 1% sodium bisulphite (50 w/v% aqueous solution, vwr chemicals, Amsterdam, the Netherlands) to prevent microbial regrowth. Sodium bisulfite was added to the feed tank and recirculated to ensure full impregnation of the membrane. If the membrane of the filtration setup needed to be removed after an experiment, the membrane filtration system was thoroughly rinsed with tap water and deionized water (dH₂O), then left open to dry. Before each treatment, the membrane filtration setup was thoroughly rinsed to remove residual bisulfite solution. If a membrane was not already installed, it was inserted into the system before the rinsing step. The rinsing procedure simultaneously flushed both the setup and the membrane under the normal operating conditions of the respective membranes. The process, which involved filling, recirculating, and draining, continued until the conductivity values of the feed, permeate, and concentrate dropped below 20 μS/cm, ensuring the system was free of contaminants. Once this criterion was met, the fractionation process could begin.

Before the treated waters were introduced into the pilot distribution system, samples were collected from the permeate and concentrate for TOC, HPSEC-TOC, and IC analyses Additionally, manual samples for flow cytometry, 16S rRNA gene-based amplicon sequencing were taken from the feed water, UF-, and NF-treated water. For the treated waters these samples were taken from the IBC in which the water was stored.

#### 2.1.2 Invasion experiment

*Aeromonas media*, *Pseudomonas putida*, and *Serratia fonticola*, were selected as bacterial indicators for the invasion experiment. They were isolated from the Flemish drinking water distribution network (Pidpa in Antwerp, De Watergroep in East–West Flanders, and Farys in Ghent, respectively) and subsequently identified with 16S rRNA gene Sanger sequencing as described by Kerckhof et al. (2021). Strains of the selected bacteria were revived from the −80°C stock and a scrape of each bacterial culture was spread on a Reasoner’s 2A (R2A) (18.1 g/L final concentration) agar plate (Oxoid, England). These plates were incubated for 24 h at 28°C. Afterward, a few colonies were selected from the agar plate, resuspended in 5 mL of 3 g/L R2A broth medium (Oxoid, England), and incubated at 28°C with shaking at 100 rpm for 24 h. Next, the cultures were washed three times using a sterile 8.5% NaCl solution. They were centrifuged for 5 min at 2500 × g, the supernatant was removed and 5 mL sterile 8.5% NaCl was added. Subsequently, the cultures were transferred to a diluted liquid medium of 50 mg/L R2A broth medium (Oxoid, England) and incubated at 28°C and 100 rpm for 24 h. Afterwards, the same washing procedure was executed again and a final 5 mL filtered 0.85% NaCl solution was added to measure the intact cell counts (ICC) using flow cytometry. The culture was diluted using sterile 8.5% NaCl solution until 10^6^ cells/mL and 1 mL of each indicator was added to each loop. Final spike concentrations ranged from 100 to 600 cells/100 mL. After 6 weeks of operation with the treated waters, the loops of the drinking water distribution system pilot were refilled with 750 L of the respective water types (L1: NF-treated water, L2: UF-treated water, L3: untreated tap water) and an invasion experiment (conducted during sampling campaign III) was performed. After 1 h of recirculation (at 0.08 m/s), samples were collected for 16S rRNA gene-based amplicon sequencing, ATP, TOC, HPSEC-TOC, and IC analysis. Subsequently, 1 mL of a solution containing 10^6^ cells/mL of each unwanted microorganism was introduced into each loop. The survival of these microorganisms was monitored for 7 days using selective plating (described in Section 2.2.2).

### 2.2 Microbial monitoring

#### 2.2.1 Bulk water phase

Online flow cytometry was applied to measure total cell concentrations and to perform phenotypic fingerprinting. To achieve continuous and automated measurements, an onCyt© (onCyt Microbiology AG, Switzerland) autosampler was coupled to an Accuri™ C6 Plus flow cytometer (BD Biosciences, Belgium) as described in Waegenaar et al. (2024b) (Supplementary Figure S1B). Briefly, samples (200 μL) were taken in triplicate for each loop every 6 h. Staining was performed using 200 μL SYBR Green I (10,000 × concentrate in DMSO, Invitrogen, Belgium), 5,000 times diluted in TRIS buffer (pH 8, 10 mM, Merck, Belgium). After mixing, the samples were incubated at 37°C for 20 min in the onCyt chambers and sent to the flow cytometer for measurement. In between measurements, cleaning of the onCyt sample lines was performed with a sodium hypochlorite solution (1 v% final concentration, Avantor, USA), after which the bleach solution was quenched with a sodium thiosulfate solution (50 mM final concentration, Merck, Belgium) and rinsed with ultrapure water (Milli-Q, Merck, Belgium). As sheath fluid, ultrapure water (Milli-Q, Merck, Belgium) was used. Manual samples, taken from the IBC in which the treated waters were stored, were collected during sampling campaign II and measured with similar staining and incubation conditions on an Accuri™ C6 Plus flow cytometer (BD Biosciences, Belgium) in the lab. This was specifically done to compare the raw treated water with the samples collected by the online flow cytometer, which monitored the water recirculated in the pilot.

Samples for 16S rRNA gene-based amplicon sequencing were taken. For sampling campaign I and III this was done on day 1 and day 7, while for sampling campaign II only on day 7. From each loop, 2 L was filtered over a 0.22 μm MCE Membrane filter (Merck, Belgium) using a filtration unit consisting of six filtration funnels and a Microsart e.jet vacuum pump (Sartorius, Germany), after which the filter was stored in a freezing tube at −21°C. Further processing for 16S rRNA gene-based amplicon sequencing (DNA extraction, PCR amplification, 16S sequencing) is described in Waegenaar et al. (2024b). In brief, DNA extraction was performed using the DNeasy PowerSoilPro kit (Qiagen, Germany), following the manufacturer’s protocol. Ten μL genomic DNA extract was send out to LGC genomics GmbH (Berlin, Germany) for library preparation and sequencing on an Illumina Miseq platform with v3 chemistry (Illumina, USA).

#### 2.2.2 Selective plating and MALDI-TOF mass spectrometry to determine bacterial indicator concentrations

The concentration of each bacterial indicator in the bulk water was determined by filtering various dilutions in triplicate (100 mL) through S-Pack filters with a pore size of 0.45 μm (Merck, Belgium). This was performed using a filtration unit consisting of six filtration funnels and a Microsart e.jet vacuum pump (Sartorius, Germany). The filters were incubated at 37°C for 18–24 h on three different types of agar media to assess the concentrations of *Aeromonas media*, *Pseudomonas putida*, and *Serratia fonticola*. Specifically, Ampicillin Dextrin Agar (ADA) (HiMedia, Germany) was used for *Aeromonas media*, Pseudomonas Cetrimide (PCN) agar (VWR Chemicals, Netherlands) for *Pseudomonas putida*, and Chromogenic Coliform Agar (CCA) (Carl Roth, Belgium) for *Serratia fonticola*. For the preparation of the PCN agar, 15 mL of glycerol (Glycerine ROTIPURAN® ≥ 99.5%, Carl Roth, Belgium) was added per 1,000 mL of agar before autoclaving at 121°C. For the ADA, after autoclaving, ampicillin (Ampicillin Dextrin Selective Supplement, HiMedia, Germany) was added once the medium cooled to approximately 50°C. The selective agars are in accordance with the ISO 9308-1:2014 method for drinking water (International Organization for Standardization, 2014). As a control measure, the bulk water of the pilot was filtered for selective plating before the addition of the unwanted microorganisms. Samples were taken 20 min (0.33 h), 1, 4, 13, 20, 42, 93, 111, and 158 h after the spike. To identify if counted indicators were the spiked indicators, random colonies obtained from selective plates were selected to analyze with MALDI-TOF MS using a Vitek MS (bioMérieux, Marcy-l’Étoile, France).

#### 2.2.3 Biofilm sampling

To sample the biofilm, coupons composed of the same material as the pipes (PVC-U), were installed in every loop (Supplementary Figure S1). As mentioned before, mature biofilm samples (*n* = 2) (coupons that were already in the pilot before) were taken before the start of the experiment (day 0), after 21 and after 42 days. Besides that, young biofilm samples (grown over the course of the experiment) were taken at day 42 (before the invasion) and day 49 (after the invasion experiment). Biofilm cells were removed using the protocol described in Waegenaar et al. (2024b). In summary, biofilm cells were removed using an electric toothbrush (Oral-B Advanced Power, Procter & Gamble, Belgium) and collected in 15 mL of 0.2 μm filtered bottled water (Evian, France). The biofilm suspensions were measured with flow cytometry using an Attune NxT BVXX flow cytometer (ThermoFisher Scientific, USA). To do so, samples were first 10 times diluted in 0.2 μm filtered bottled water (Evian, France). Staining was done with 1 v% of 100 times diluted SYBR Green I (10,000 × concentrate in 0.22 μm-filtered DMSO, Invitrogen, Belgium) solution to measure total cell counts (Waegenaar et al., 2023). Staining was also done with 1 v% of 100 times diluted SYBR Green I combined with propidium iodide (10,000 × concentrate in 0.22-μm filtered DMSO, 50 × 20 mM propidium iodide in 0.22-μm filtered DMSO, Invitrogen, Belgium) to measure intact-damaged cells. Incubation was done at 37°C for 20 min in the dark. All samples were measured in technical quadruplicate. In addition, 3 mL (3×) of each suspension was filtered using S-Pack filters with a pore size of 0.45 μm (Merck, Belgium). The filters were then placed on the three different types of agar media to assess the concentrations of *Aeromonas media*, *Pseudomonas putida*, and *Serratia fonticola*. The remaining volume was filtered using MCE Membrane filters (Merck, Belgium) and Polycarbonate syringe filter holder (Sartorius, Germany) to perform 16S rRNA gene-based amplicon sequencing.

### 2.3 Chemical monitoring

#### 2.3.1 Total organic carbon analysis

Samples for TOC analyses were collected in 40 mL TOC-free vials (Sievers, Germany) and stored at 6°C prior to analysis. TOC concentrations were measured in technical quadruplicate using a Sievers 900 Portable TOC Analyzer connected to a Sievers 900 Inorganic Carbon Remover (General Electric Company, Boston, USA).

#### 2.3.2 Size exclusion chromatography with total organic carbon detection

High performance size exclusion chromatography was used to determine the concentration of high MW (MW > 20.3 kDa), medium MW (MW between 20.3 kDa and 0.286 kDa) and low MW (MW < 0.286 kDa) fractions in the water samples, using the method described in Laforce et al. (2024). Samples were taken at the start of each sampling campaign, collected in 40 mL TOC-free vials (Sievers, Germany) and stored at 6°C before analysis within 2 weeks.

#### 2.3.3 Ion chromatography

Samples for anions (NO_3_^−^, NO_2_^−^, SO_4_^2−^, Cl^−^) and cations (Ca^2+^, Mg^2+^, K^+^, Na^+^) were collected in reusable IC tubes (PROMED, Italy), that were rinsed with ultrapure water (Milli-Q, Merck, Belgium), and stored at 6°C prior to analysis. The ions were separated using ion exchange chromatography (IC) by an 930 Compact IC Flex (Metrohm, Switzerland). The device is equipped with a Metrosep A Supp 5150/4.0 column and a Metrosep A Supp 4/5 guard column/4.0, to protect the column from contamination, and 850 IC conductivity detector (Metrohm, Switzerland). As the mobile phase, a 1.7 mM HNO_3_ (2 M, ThermoFisher Scientific, USA) and a 1.7 mM 2,6 pyridinedicarboxylic acid solution (Merck, Belgium) was used for the elution of the cations and a 1.0 mM NaHCO_3_ (≥99.5%, Carl Roth, Germany) and 3.2 mM Na_2_CO_3_ (≥99.5%, Carl Roth, Germany) solution was used for the elution of the anions.

### 2.4 Data analysis

Data analysis was done in R (R Core Team, 2022) in RStudio version 4.3.0 (RStudio Team, 2020). The Flow Cytometry Standard (.fcs) files were imported using the flowCore package (v2.14.0) (Ellis et al., 2022). The background data was removed by manually drawing a gate on the FL1-H (green) and FL3-H (red) fluorescence channels as described in Props et al. (2016). Illumina data was processed using the DADA2 pipeline (v1.30.0) (Callahan et al., 2016, p. 2). Taxonomy was assigned using the Silva database v138 for the 16S rRNA gene-based amplicon sequencing (Guillou et al., 2012; Quast et al., 2013). For 16S rRNA gene-based amplicon sequencing normalization of the sample reads was done to correct for differences in sequencing depth among samples. The sequencing reads were ranging from 4,533 to 126,092. Further data analysis was performed using packages such as the phyloseq package (v1.46.0) and the vegan package (v2.6–4) (McMurdie and Holmes, 2013; Oksanen et al., 2022). The data generated by MALDI-TOF MS was analyzed using the MYLA® software (Pidpa, Antwerp). Data visualization was done using the ggplot2 (v3.4.4) and ggpubr (v0.6.0) packages (Kassambara, 2020; Wickham, 2016). Shapiro–Wilk Test was used to test the data for normality. Further statistical analysis was done using the vegan package for ANOSIM tests (v2.6–4), and base R’s stats package functions kruskal.test() for Kruskal-Wallis, wilcox.test() for Mann–Whitney, and aov() for one-way ANOVA analyses (Oksanen et al., 2022). If numbers are reported as X ± Y, Y is the standard deviation. If only ±X is shown, X is the average.

To calculate the cation, anion and TOC rejection efficiencies, the concentration of the ion in the permeate stream (C_p_) was deducted from the concentration in the feed stream (*C_f_*) and then divided by the concentration in the feed (*C_f_*) (Equation 1). This number was then multiplied by 100 to show the final result in percentage.


(1) $$\text{Rejection efficiency}=\frac{C_{f}-C_{p}}{C_{f}}\times 100$$

To evaluate the decay of each unwanted microorganism during the invasion experiment, first-order decay rate constants (k (h^−1^)) were calculated as the slope of the line when ln(C_t_/C_0_) was regressed against time (t), where C_t_ is the concentration of the concerned microorganism (CFU/100 mL) at a certain time t and C_0_ is the concentration of the concerned microorganism (CFU/100 mL) at time 0 (Equation 2) (Chick, 1908).


(2) $$C_{t}=C_{0}*e^{-\mathrm{kt}}$$

## 3 Results

### 3.1 Evaluation of the membrane filtration efficiencies

**Figure 2:**
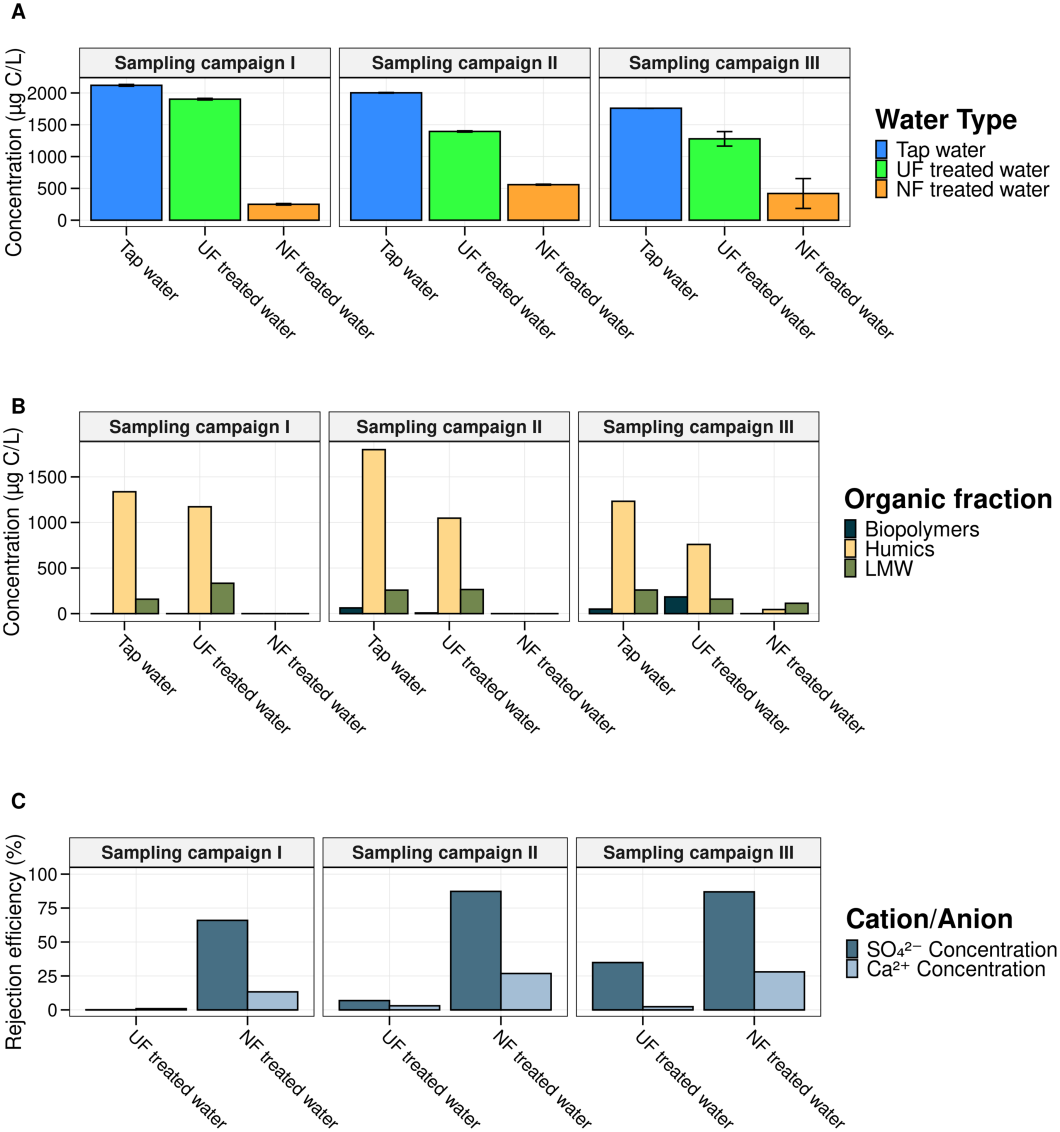
Overview of the chemical analysis of the NF-, UF-, and untreated tap water for each sampling campaign. **(A)** TOC concentration in μg/L (*y*-axis) for each of the water types (*x*-axis). Per water type four technical replicates were measured and the associated error bars are shown in black. **(B)** The concentration (μg/L) of the low, medium and high molecular weight fraction measured using HPSEC-TOC for each of the water types (*x*-axis). **(C)** The respective rejection efficiencies (*y*-axis) based on the results of the IC analysis for SO_4_^2−^ and Ca^2+^ for the NF and UF treated water. The rejection efficiencies were calculated using Equation 1. These results demonstrated the efficient removal of bivalent cations/ions for NF.

To evaluate the membrane filtration efficiency, TOC, HPSEC-TOC, and IC analysis were performed for the three different sampling campaigns during the 7-week experiment (Figure 1). Samples were taken from the UF- and NF-feed, the UF- and NF-treated water, and the tap water used for the control loop. Overall, we showed that the UF and NF treatment were successful, as the treated waters showed a decrease in overall TOC levels and chemical compounds (Figure 2). Untreated tap water had a TOC concentration of 1960.833 ± 150.08 μg/mL (*n* = 12). For UF, a TOC concentration of 1463.75 ± 268.58 μg/mL (*n* = 12) was measured, meaning that 435.42 μg/mL of TOC was removed compared to the tap water. NF removed approximately three times more TOC (±1478.50 μg/mL), as the NF-treated water had an average TOC concentration of 482.33 ± 167.96 μg/mL (*n* = 12). This resulted in an average TOC rejection efficiency of 75.40% for NF and 25.35% for UF (Equation 1). The HPSEC-TOC results showed that NF is capable of removing all organic fractions below the detection limit, including the high MW (MW < 20.3 kDa), medium MW fraction (MW between 0.286 kDa and 20.3 kDa), and low MW compounds (MW < 0.286 kDa), whereas this was not the case for the UF treatment. The IC results revealed more differences between UF and NF, with NF demonstrating higher removal efficiencies for bivalent anions (SO₄^2−^) and cations (Ca^2+^), averaging 5.75 and 10.90 times higher than UF across all sampling campaigns, respectively, which is in line with the characteristics and expectations for UF and NF.

Furthermore, the membrane filtration techniques influenced the microbial traits of the treated waters, as flow cytometric measurements confirmed microbial removal and filtration efficiency, with both for NF and UF measurements approaching the detection limit of 1,000 cells/mL. Additionally, NF- and UF-treated water, as well as untreated waters were characterized by 16S rRNA gene-based amplicon sequencing, which demonstrated that the UF- and NF-permeate had a similar microbial composition (Supplementary Figure S5). This indicates that both filtration processes removed certain bacteria, resulting in a distinct microbial community introduced into the pilot system.

### 3.2 Microbial water quality during distribution when introducing NF and UF tap water

**Figure 3:**
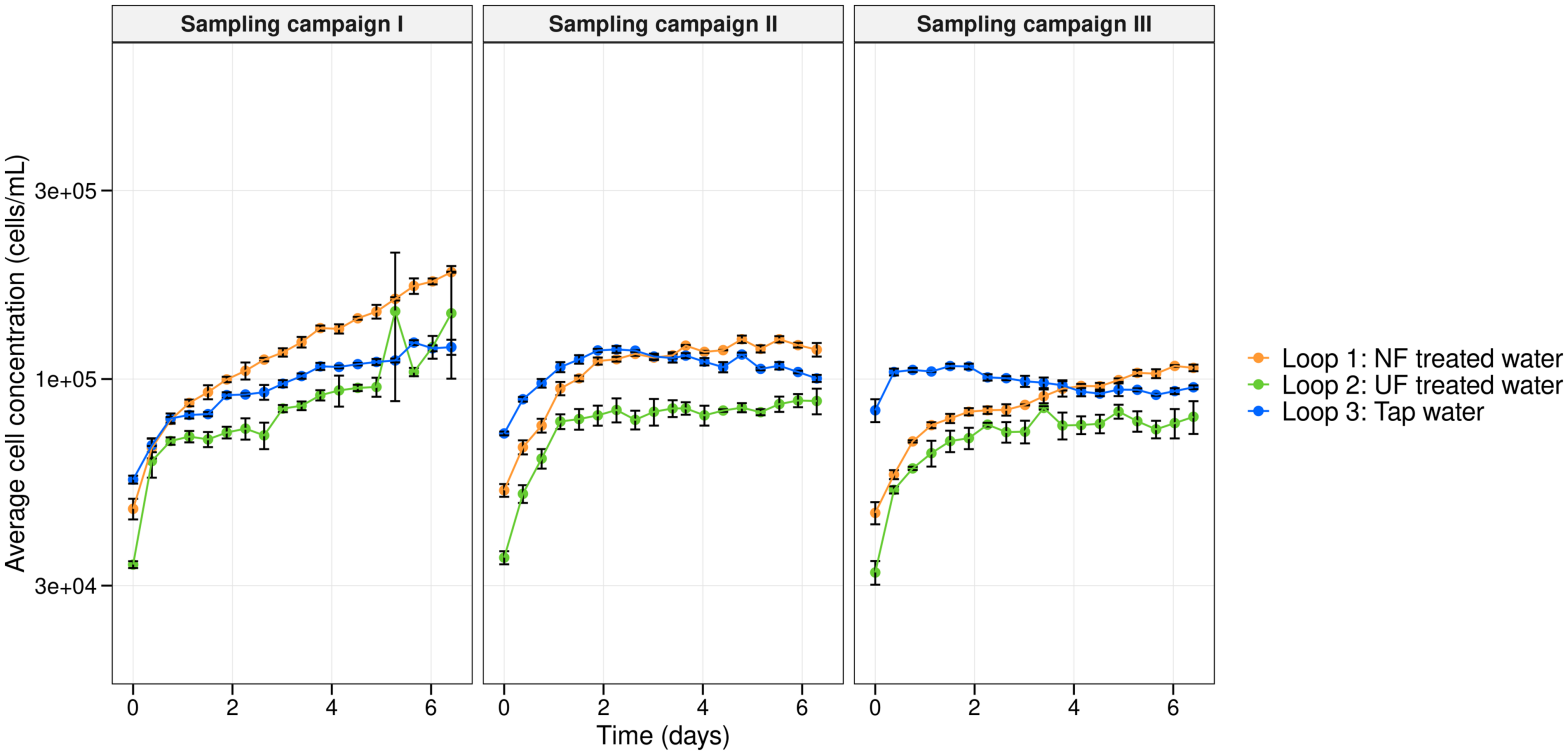
Average cell concentration (cells/mL) in function of time (days) of the microbial bulk community in loop 1 (NF-treated water) (orange), loop 2 (UF-treated water) (green), and loop 3 (tap water) (blue) for each sampling campaign. Per timepoint, biological replicates (*n* = 3) were taken and corresponding error bars are shown in black.

Over the course of 7 weeks, the DWDS pilot was supplied weekly with treated water: NF-, UF- treated tap water to loops 1 and 2, respectively, and untreated tap water to loop 3 (control) (Figure 1). Using online flow cytometry the microbial cell concentrations of the bulk water phase were monitored for three sampling campaigns that were carried out in the 7-week experiment (Figure 3). Despite differences in initial cell concentrations, with a lower cell concentration for the loops that received UF- and NF-treated water (the average cell concentration of the first measurements of the three sampling campaigns (*n* = 3) for loop 1, 2 and 3, was, respectively, 4.8 × 10^4^ ± 2.8 × 10^3^, 3.4 × 10^4^ ± 1.2 × 10^3^, and 7.1 × 10^4^ ± 1.1 × 10^4^ cells/mL), all of the loops showed increasing cell concentrations throughout each sampling campaign. Though, there was a significant difference for the cell concentrations between the three loops (between L1, L2 and L3) for sampling campaign I and sampling campaign III (Exp I and Exp III: *p* < 0.05, Kruskal–Wallis chi-squared). Only in sampling campaign II, there was no significant difference between the loop with tap water (loop 3) and the loop recirculated with NF-treated water (loop 1) (*p* = 0.1559, Kruskal–Wallis chi-squared). It is however important to note that despite the significant differences, all cell concentrations vary between a minimum of 2.7 × 10^4^ and a maximum of 3.1 × 10^5^ cells/mL, meaning that between the extremes of the entire experiment there was only 1-log difference, which is not much microbiologically speaking. This indicates that within and between the loops, although statistically significant, the differences in cell concentrations were minor. For sampling campaign II, additional samples were taken manually from the fresh UF- and NF-treated waters and tap water, before they were introduced into the pilot. These samples were analyzed with flow cytometry. In the NF treated water, UF treated water, and tap water, cell concentrations of 1.8 × 10^3^, 1.0 × 10^3^, and 1.0 × 10^4^ cells/mL were detected, respectively. These are values that are close to the detection limit of the flow cytometer. When comparing these manual measurements with the first cell concentrations that were measured in sampling campaign II after recirculation in the pilot system (loop 1 (NF-treated water): 3.5 × 10^4^, loop 2 (UF-treated water): 5.2 × 10^4^, and loop 3 (tap water), 7.3 × 10^4^ cells/mL (measured by the online measurements of the Oncyt coupled to the Accuri C6 plus flow cytometer)), it is clear that introduction into the loops resulted in an increase in cell concentrations of, respectively, 3.32 × 10^4^, 5.1 × 10^4^, and 6.3 × 10^4^ cells/mL for loop 1 (NF-treated water), loop 2 (UF-treated water), and loop 3 (tap water).

**Figure 4:**
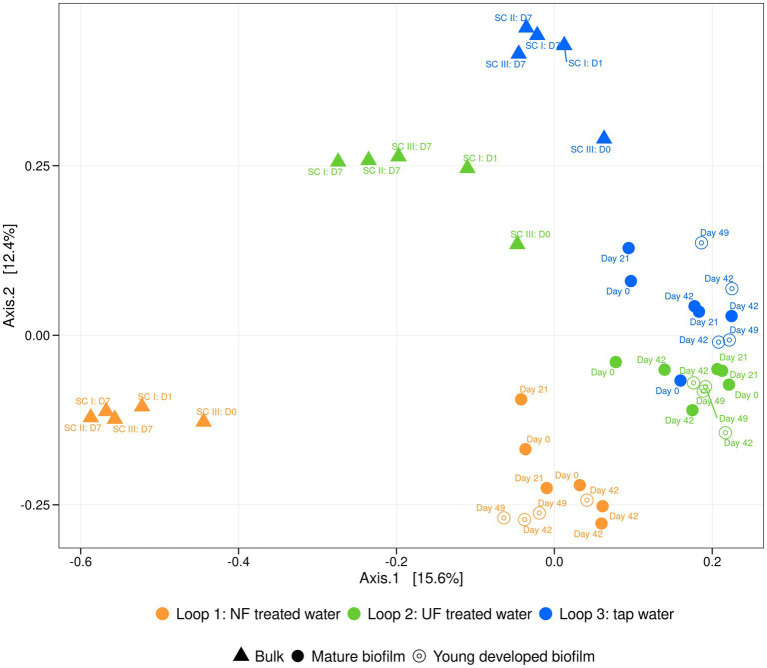
PCoA of the relative bacterial community composition of bulk (▲), the existing, mature biofilm samples (●) and the young biofilm, developed during the experiment (only exposed to treated waters) (⊙) samples of loop 1 (NF-treated water) (orange), loop 2 (UF-treated water) (green) and loop 3 (tap water) (blue). Sampling timepoints are indicated above each shape.

16S rRNA Sequencing of the raw fractions showed that the filtration altered the microbial community composition in the NF and UF treated water, in comparison to the tap water (Supplementary Figure S5). The microbial community composition of the NF and UF treated waters were similar, but when introduced into to the pilot (measurements were done after initial introduction into the system and after 7 days) the composition changed, and a distinct community was observed for each of the loops. In loop 1, which was recirculated with NF treated water, the microbial community closely resembled that of the raw NF treated water and remained stable after 7 days of recirculation, with the families *Spinghomonadaceae*, and *Commamonadaceae* being the most abundant in general. For the other loops, the community changed when it was added to the pilot and specific families (i.e., for loop 2 (UF treated water) the order *Rhodospirillales*, for loop 3 (tap water), the *SAR324 clade*) were enriched during recirculation. Based on the relatively high amount, 50%, of “others” (bacteria that are not amongst the top 20 most abundant ASV’s) it is clear that in the loop that received UF-treated water (loop 2) and the loop that received tap water (loop 3) there was a greater diversity compared to the loop that received NF-treated water (loop 1). Based on these 16S rRNA sequencing results, a principal coordinate analysis was performed, visualizing the dissimilarities (Bray-Curtis) between the different samples (Figure 4). We observed three different groups representing each loop. This indicated that the water that the loops received influenced the bulk community composition. Over time, across all different sampling campaigns (SC I, SC II, SC III), the community did not change, as the samples clustered more or less together per loop.

### 3.3 Influence of UF and NF treated tap water on biofilm stability

Before the start of the experiment (baseline), in between sampling campaign I and II (after 21 days), and at the end of sampling campaign II (after 42 days), the biofilm cell density and community composition were assessed to determine whether the treated waters influenced a mature biofilm. Next to this, at the end of sampling campaign II and III (after 42 and 49 days, respectively) young biofilm samples were collected that had only been exposed to the treated waters. For the mature biofilm samples, total cell densities remained consistent across the sampling points, ranging from 0.51 × 10^6^ to 6.10 × 10^6^ cells/cm^2^ on day 0 and from 0.49 × 10^6^ to 1.31 × 10^6^ cells/cm^2^ at the end of sampling campaign II (Supplementary Figure S7). There was no significant effect of the treated waters on the total cell density of the mature biofilm in each loop (*p* = 0.456, *p* = 0.0415; *p* = 0.682, for loops 1, 2, and 3, respectively, Mann–Whitney test). Similarly, the community composition on day 21 and at the end of sampling campaign II remained comparable to the baseline (day 0) in each loop (Supplementary Figure S8), indicating that the treated water with lower organic content had no significant impact on the mature biofilm community (*p* = 0.4, *p* = 0.133, *p* = 0.7333, for loop 1, 2, and 3, respectively, ANOSIM).

For young biofilms exposed solely to the UF and NF treated waters, total cell densities were comparable to those of the mature biofilm (present 100 days before the actual experiment), ranging from 0.50 × 10^6^ to 3.35 × 10^6^ cells/cm^2^ (Supplementary Figure S7). However, a significant difference in cell density was observed between the biofilms formed during the experiment and the mature biofilms for loop 1 that received NF-treated water (*p* < 0.05, Mann–Whitney test), but not for loops 2 (UF-treated water) and 3 (tap water) (*p* = 0.5, *p* = 0.24, for loops 2 and 3 respectively, Mann–Whitney test). Cell densities of the mature biofilms of the loop that received NF-treated water were slightly higher ((1.01 ± 0.50) × 10^6^ cells/mL) compared to the developed biofilm at the end of sampling campaign II and at the end of sampling campaign III ((0.59 ± 0.05) × 10^6^ cells/mL). Despite these differences, no significant differences in community composition across sampling points were observed between mature and the young biofilms for each loop (*p* = 0.064, *p* = 0.446, *p* = 0.135, for loops 1, 2 and 3, respectively, ANOSIM). The dominant community members in both biofilm types belonged to the Phylum *Proteobacteria*, specifically the families *Sphingomonadaceae*, *Comamonadaceae*, *Xanthobacteraceae*, and *Rhodobacteraceae*, as well as the phylum *Verrucomicrobiota*, specifically the family *Parachlamydiaceae* (Supplementary Figure S8).

Our results suggest that despite an overall similarity in biofilm community composition, underlying changes were occurring when water with the lowest organic carbon content was added to the system (Figure 4). To further investigate this, dissimilarity indices between the bacterial community of the bulk water and biofilm samples for both mature and developed biofilms throughout the experiment were calculated (Table 1). Higher dissimilarity values were observed in loop 1 (NF-treated water) compared to the other loops, meaning that the bacterial community of the biofilm and bulk of loop 1 (NF-treated water) were less similar than in the other loops. This can be confirmed by looking at the bulk community composition of the loop that received NF-treated water (loop 1), which was more different than the other loops (loop 2; UF-treated water and loop 3; tap water) (Figure 4 and Supplementary Figure S5).

**Table 1:** Dissimilarity measures calculated using 16S sequencing data at the ASV level (Bray-Curtis based on relative abundances).

	Bulk vs. mature biofilm	Bulk vs. developed biofilm	Mature biofilm vs. Developed biofilm
Loop 1 (NF-treated water)	0.90	0.90	0.62
Loop 2 (UF-treated water)	0.81	0.86	0.74
Loop 3 (tap water)	0.82	0.83	0.63

For the mature biofilm samples, data from day 21 and day 42 were considered, along with bulk water samples collected after a 7-day in SC II and III. For the young developed biofilm, samples from day 49 and bulk water samples from SC III were analyzed.

### 3.4 Invasion potential of unwanted microorganisms

**Figure 5:**
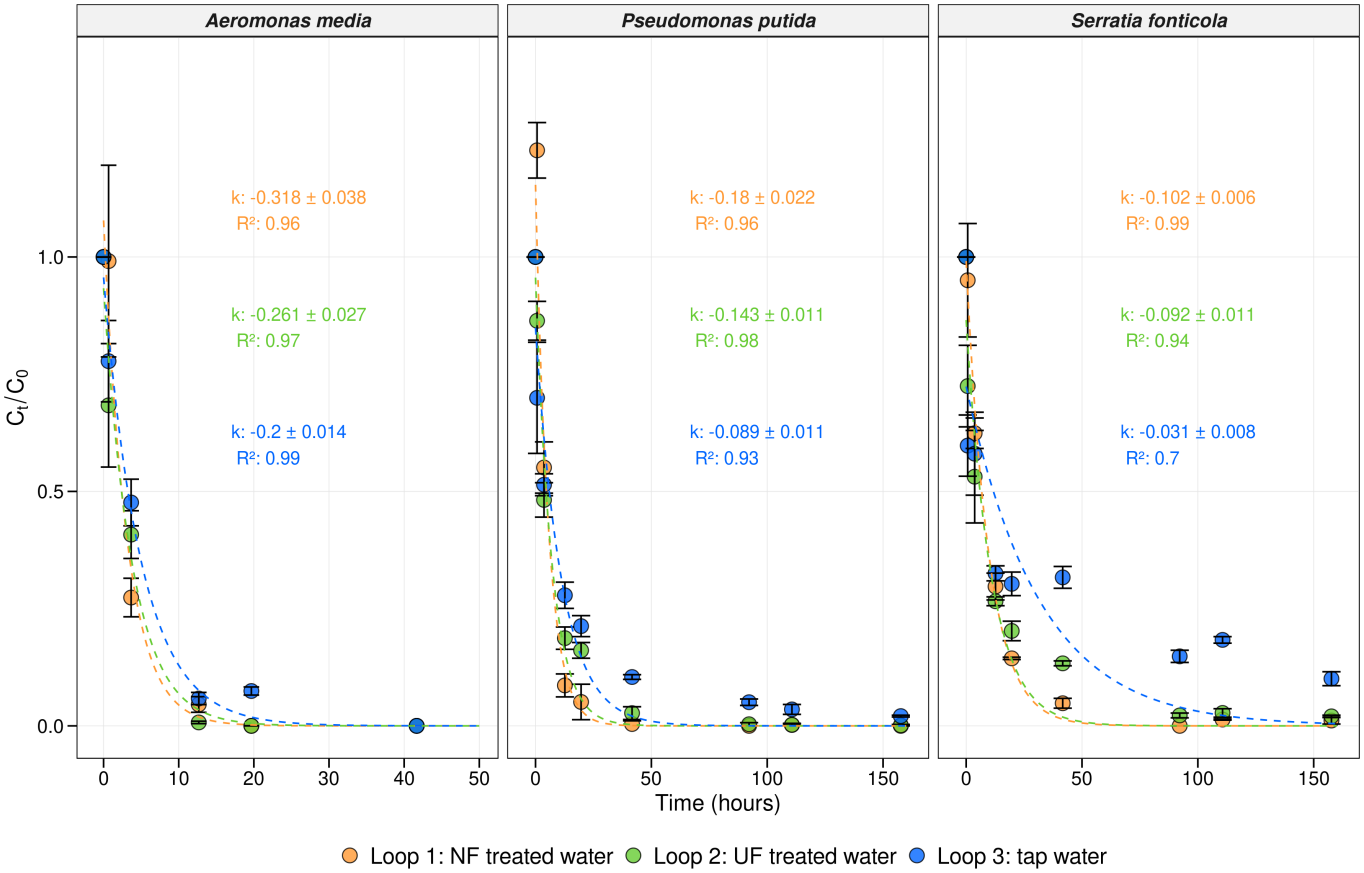
Concentration and decay rates of the bacterial indicators. Average concentration in function of time (hours) for each bacterial indicator at each temperature scenario in loop 1 (NF-treated water) (orange), loop 2 (UF-treated water) (green), and loop 3 (tap water) (blue). The average concentration was calculated as $C_{t}/C_{0}$ where $C_{t}$ represents the average concentration at time t, and $C_{0}$ the average concentration at the initial timepoint (t = 0). Per timepoint, biological replicates (*n* = 3) were taken and corresponding error bars are shown in black. First-order decay rate constants (k (h^−**1**^)) were calculated using Equation 2. The model predictions, along with their respective R squared values, are depicted for each loop with corresponding colors, represented by dotted lines.

To evaluate the invasion potential of unwanted microorganisms in water with a lower organic carbon load, an invasion experiment was performed during sampling campaign III (Figure 1). One mL of a solution containing 10^6^ cells/mL of *Aeromonas media*, *Pseudomonas putida*, and *Serratia fonticola* was introduced into each loop in order to achieve a final concentration of 100 CFU/100 mL of each microorganism. The survival of the bacterial indicators was followed for 7 days using membrane filtration and selective plating techniques. Additionally, first-order decay rate constants (k (h^−1^)) were calculated using Equation 2, with higher k values indicating more rapid decay of the corresponding microorganism (Figure 5). The initial concentrations were ranging between 170 CFU/100 mL to 800 CFU/100 mL, aligning with the target concentration of 100 CFU/100 mL (Supplementary Figure S9). *A. media* was no longer detected in any loop 30 h after the spike, while *P. putida* and *S. fonticola* persisted for 150 h. This was confirmed by the decay rates of *A. media* which were higher than those of the other microorganisms. In addition, a faster decay of each microorganism was observed in loop 1, (NF treated water), and in loop 2 (UF treated water), compared to loop 3 (untreated tap water). For *A. media*, a significant difference was observed between loop 1 and the other loops (*p* < 0.05, Tukey test), while no significant difference was found between loops 2 and 3 (*p* = 0.26, Tukey test) after 4 h. For *P. putida* and *S. fonticola*, significant differences between the loops emerged after 12 h (*p* < 0.05, one-way ANOVA). This was further supported by the first-order decay rate constants, which were lower in loop 3 (tap water) than loops 1 (NF-treated water) and 2 (UF-treated water). For example, the decay rate for *P. putida* in loop 3 was −0.089 h^−1^, while in loops 1 and 2, it was 0.18 h^−1^ and -0.143 h^−1^, respectively (Figure 5). These findings suggest that lower TOC levels and the removal of biopolymers, part of the humic substances and low molecular weight compounds result in a faster decay of unwanted microorganisms.

## 4 Discussion

### 4.1 Nutrient limitation altered bulk community composition, but microbial cell concentrations remained similar

Over the course of 7 weeks, NF- and UF-treated water was introduced into a pilot-scale DWDS with a mature biofilm (consistent cell concentrations and equilibrium between growth, attachment and detachment; Boe-Hansen et al., 2002b; Waegenaar et al., 2024b) to investigate the impact of nutrient limitation on microbial (re)growth and its influence on both mature biofilms and biofilm development. Using TOC, HPSEC-TOC, and IC, the treated water quality and nutrient removal efficiencies were evaluated, confirming expected removal efficiencies across different treatments (Figure 2). The TOC results for UF- and NF-treated water align with previous studies, as UF showed little reduction in TOC, whereas NF removed nearly all TOC (Rehman et al., 2020). Similar TOC removal efficiencies for NF membrane filtration as in our sampling campaigns (±75.40%) were found in previous studies (Fan et al., 2020; Yuksekdag et al., 2023). Fan et al. (2020) and Yuksekdag et al. (2023) reported TOC removal efficiencies for the NF treated water of 96.0% and after dual UF-NF treatment 89.21%, respectively. In a study by De La Rubia et al. (2008) several NF membranes were studied and showed removal efficiencies ranging between 75% and 90% (De La Rubia et al., 2008).

In addition, NF resulted in an almost complete removal of all organic fractions (Laforce et al., 2024) (Figure 2). Assimilable organic carbon (AOC) primarily consists of low MW compounds(Dejaeger et al., 2025; Rehman et al., 2020) and is considered to be one of the most important factors for microbial regrowth in DWDS (Hammes et al., 2009; Polanska et al., 2005; Song et al., 2021). Granting that in our sampling campaigns AOC was not directly measured, HPSEC-TOC measurements demonstrated that the low MW compounds were effectively removed (Figure 2B). In previous research, it was shown that NF (ESNA1- LF-4040 Spiral wound membrane, 7.9 m^2^ membrane area, nominal CaCl_2_ rejection of 91%) removes 52% of AOC, and although it is believed that chlorination is still required to achieve biostability, it can be applied at lower concentrations in comparison to systems with no AOC removal (Ohkouchi et al., 2014). So, given that previous full-scale and pilot-scale studies have demonstrated that, although significantly removed, AOC is still found in the NF treated water (MWCO of 200 Da, which is slightly lower than the MWCO of 300–500 Da of the membrane used for our experiment), and in such quantities to still cause (re)growth, it is likely that there was still AOC present in our system and experiments (Escobar et al., 2000; Escobar and Randall, 2001, 1999; Park et al., 2005; Zhou et al., 2009). This consideration is crucial, as AOC is a key indicator of biostability (Hammes et al., 2009; Hem, 2001; Prest et al., 2016; Srinivasan and Harrington, 2007). Besides AOC, other nutrients like phosphate and nitrogen also influence microbial re(growth), though, they were not directly measured here (Morton et al., 2005; Yousefi et al., 2016). NF membranes (MWCO 200 Da) effectively remove phosphate, unlike conventional UF membranes (Escobar and Randall, 2001; Gao et al., 2019). However, since the feed water was already low in phosphate, differences were likely minimal and had limited impact on bulk water and biofilm development (Waegenaar et al., 2024b).

Before it was introduced in the pilot-scale DWDS, microbial cell concentrations of the NF- and UF- treated water were close to the detection limit of the flow cytometer. Once introduced in the pilot, the microbial cells in the bulk water phase increased over time (Figure 3). In previous experiments done by Chan et al. (2019), a reduction of around 93.0% in bacterial cell counts was seen for UF-treated water. For sampling campaign II, the raw fractions were measured using flow cytometry and a reduction in cell concentration of 81.7 and 89.5% for NF and UF, respectively, was observed compared to the number of cells in the feed (tap water). Although this is lower than would be expected for UF and NF treatment, it is important to highlight that in this experiment the feed water that was used was already treated tap water containing 1.0 × 10^4^ cells/mL, which is lower than what is regularly found in surface water (untreated water) for example (i.e., Albert Canal (Belgium): 1.0 × 10^6^ – 1.0 × 10^7^ cells/mL), thus explaining the relatively lower log reduction value. Once introduced and recirculated into the pilot-scale DWDS, the cell concentrations increased to 3.32 × 10^4^, 5.1 × 10^4^ and 6.3 × 10^4^ cells/mL for loop 1 (NF-treated water), loop 2 (UF-treated water), and loop 3 (tap water), respectively (Figure 3). A previous full-scale study has shown that biofilm detachment in nutrient limited water (UF) could cause a 1 log increase in cell concentrations in the bulk, similar to what we have seen (Chan et al., 2019). Several other studies have described that under nutrient limiting conditions dispersal related genes are expressed, causing biofilm bacteria to detach (De Plano et al., 2024; Díaz-Salazar et al., 2017; Nair et al., 2021). However, since a similar rise in cell concentrations was also observed in the control loop (loop 3; tap water), this increase, and possible biofilm detachment, cannot be solely linked to the addition of nutrient-limited water. Additionally, biofilm detachment should be interpreted with caution, as Bray–Curtis dissimilarity indices show clear differences between bulk water and biofilm communities (Table 1). If significant exchange between the biofilm and bulk phase had occurred, these dissimilarities would be expected to be lower. For the loop with NF-treated water (loop 1), the community composition of bulk and biofilm samples was more different compared to the differences between bulk and biofilm of the other loops (Figure 4 and Table 1). Therefore, detachment from the biofilm induced by the low nutrient environment seems less likely. In a study by Sibille et al. (1997) in which NF was added to a distribution system, no sloughing of the biofilm and an increase in bulk bacteria during distribution was seen, similar to our results.

Based on flow cytometric cell concentrations and 16S rRNA gene-based amplicon sequencing results (Figures 3, 4 and Supplementary Figures S4, S5) of the raw fractions, it is evident that the filtration treatment of UF- and NF- treated water influenced both bulk cell abundance and community composition. The observed increase in microbial counts in the pilot system (Figure 3) may, instead of biofilm detachment, be attributed to the use of non-sterile tubing and IBCs for treated water collection, or the presence of residual AOC, phosphate, and nitrate remaining after membrane filtration in the fractions and/or IBCs (Kantor et al., 2025; Pluym et al., 2023). Additionally, based on the small differences in cell concentrations (within each loop) and the minor differences that were seen in the community composition between the beginning and the end of each week (Figure 3), it cannot be considered to be (re)growth in the pipes, but rather be caused by bacteria that were already present. All three loops, however, reached a similar cell concentration after 7 days of recirculation (Figure 3). Previous studies have demonstrated that under low-nutrient conditions, bulk phase bacteria exhibit higher specific activity than biofilm-associated bacteria, with bulk phase growth contributing significantly to overall regrowth in drinking water systems (Boe-Hansen et al., 2002a). Since each loop’s bacterial community contained specific taxa, the growth observed during the recirculation phase may be attributed to the selection of microorganisms favoring lower organic carbon concentrations in the respective treated waters. However, it important to mention that the pilot distribution system is a recirculation system, operated at a fixed pressure, temperature, and flow regime. It was designed to closely mimic operational conditions, and the experiments were ultimately tested under ideal and stable circumstances. As such, while some outcomes may be generalized, results could vary across different systems and contexts.

### 4.2 Treated water left the mature biofilm unaffected, while NF led to lower cell densities in the young biofilm

In addition to bulk water analysis, biofilm samples were taken before the start of the experiment (baseline), in between sampling campaign I and II (after 21 days), and at the end of sampling campaign II (after 42 days). Furthermore, at the end of sampling campaign II and III (after 42 and 49 days, respectively) young biofilm samples were collected that had only been exposed to the treated waters. The biofilm cell density and community composition were assessed to determine whether NF- and UF-treated water influenced a mature biofilm, as well as to determine whether treated water conditions support the development of biofilms (Figure 4 and Supplementary Figures S6, S7). Before the addition of treated water, the biofilm in the pilot-scale DWDS was cultivated for 100 days, resulting in a stable and mature biofilm (Boe-Hansen et al., 2002b; Waegenaar et al., 2024b). This stable biofilm was not influenced by the addition of NF- and UF-treated water, as community composition nor cell density were affected (Figure 4 and Supplementary Figures S6, S7). The PCoA analysis based on the 16S rRNA gene-based amplicon sequencing results confirmed that the composition of the mature biofilm remained largely unchanged throughout the experiment in each loop (Figure 4). Biofilm samples from the same loops consistently grouped together over time, suggesting that prolonged exposure to NF- and UF-treated tap water did not significantly altered the biofilm’s community composition.

Next, to influence of the treated water on a mature biofilm, the cell densities and community composition of young biofilms that were only exposed to the treated waters were evaluated, as it is hypothesized that nutrient limitation (mainly carbon), is a way to control biofilm development (Liu et al., 2016). Cell densities of the young biofilm were comparable to the cell densities of the mature biofilms, however, the cell densities in loop 1, after NF addition, were slightly but significantly lower compared to the mature biofilm samples of the same loop (Supplementary Figure S7). In contrast, the community composition of the young biofilms was similar and not significantly different to the mature biofilms (Supplementary Figure S8). In studies from Boe-Hansen et al. (2002b, 2002a), they have determined biofilm growth under nutrient limiting conditions (AOC < 5 μg-C/L) with a net growth rate of 0.013 day^−1^ and a stationary bacterial cell density of 2.6 × 10^6^ cells/cm^2^. These results are in line with the observed cell density of loop 2 (UF-treated water) and 3 (tap water) in our experiment but 0.5 log higher than the cell density of loop 1 (NF-treated water). Besides nutrient limitation of the bulk water, it has been seen that biofilm growth is driven by the organic carbon migrating from the polymeric pipe material (Bucheli-Witschel et al., 2012; Neu et al., 2019). So, since our pilot consists out of PVC piping, biofilm formation might be mostly driven by the nutrients leaching from the pipe material, next to the nutrients from the bulk water. Additionally, the similarity in bacterial community composition of the young biofilms compared to the mature biofilm samples, despite differences in the bulk water community, may suggest that colonization is primarily driven by surrounding biofilm-associated bacteria.

### 4.3 Faster decrease of unwanted microorganisms in water with less organic carbon

After 6 weeks of weekly additions of NF-, UF-, and non-treated waters to the distribution pilot, an invasion experiment was performed in the seventh week (sampling campaign III) with *Aeromonas media*, *Pseudomonas putida*, and *Serratia fonticola* (Figure 1) to determine the survival of unwanted microorganisms in water with lower organic carbon levels (Figure 5). The initial concentrations of each indicator organism in each loop was approximately 100 CFU/100 mL (Supplementary Figure S9), which was 10^5^ times lower than spike concentrations used in previous studies, in order to simulate realistic contamination levels (Amanidaz et al., 2015; Bivins et al., 2017; Lehtola et al., 2007; Moritz et al., 2010; Park et al., 2024; Van Nevel et al., 2013; Waegenaar et al., 2024a). The survival of the unwanted microorganisms was followed for 7 days using membrane filtration and selective plating techniques. In general, a decrease of each microorganism was observed over time, with *A. media* exhibiting a faster decrease compared to *P. putida* and *S. fonticola* (Figure 5). *A. media* was no longer detected in any loop 30 h after the spike, while *P. putida* and *S. fonticola* persisted for 150 h. Similar findings were obtained in a study from Waegenaar et al. (2025). These decreases were confirmed by first-order decay rate constants, calculated according to the Chick equation (Equation 2). The decay rate was found to be higher for *A. media* compared to the other indicator organisms, being up to 10 times higher in loop 3 (control loop; tap water) and twice as high in loops 1 and 2, which were fed with NF- and UF-treated tap water, respectively (Figure 5). In loop 3, which was fed with untreated tap water, the results were similar to decay values reported in a study by Waegenaar et al. (2025). Additionally, the decay values of *P. putida* and *S. fonticola* in loop 3 were comparable to previously reported decay values for total coliforms and *Escherichia coli* in sewage and river water (Easton et al., 2005; Medema et al., 1997).

For each invader, decay patterns were faster in loops 1 (NF-treated water) and 2 (UF-treated water) compared to loop 3 (tap water). This was confirmed by the first-order decay rate constants, which were highest in loops 1, and 2, which received NF- and UF-treated water, respectively, followed by loop 3, which received tap water, suggesting a faster decay in treated water with lower organic carbon levels. It was previously shown that the bacterial invasion potential of unwanted microorganisms in water sources is determined by nutrient availability and the resident drinking water community (Van Nevel et al., 2013). In invasion experiments with *P. putida* in tap water, the addition of organic carbon prolonged the survival of *P. putida* (Van Nevel et al., 2013). Furthermore, not only was the total organic carbon content lower in the treated waters, but the biopolymer fraction was also removed. Moreover, humic substances were lower in UF waters, while both humic substances and low-molecular-weight compounds were almost completely removed in NF-treated waters (Figure 5). Hijnen et al. (2018) showed that high MW organic carbon compounds, which mainly consists of biopolymers such as carbohydrates and proteins, are correlated with regrowth (higher HPC counts), and the detection of *Aeromonas* spp., coliforms, and invertebrates during distribution. In addition, these high MW OC determine the microbial growth potential of treated water (Schurer et al., 2022). By using UF or NF techniques, high MW fractions and AOC levels are reduced (Schurer et al., 2019), enhancing overall the biostability of water.

Our results fit within the r/K strategist concept, where the unwanted microorganisms or indicator organisms act as r-strategists, characterized by a high growth rate at high nutrient concentrations, whereas the natural drinking water community consists out of K-strategists characterized by high substrate affinity (Favere et al., 2021; Vital et al., 2012). We observed that the unwanted microorganisms decreased more rapidly and struggled in water with lower organic carbon levels (Figure 5), whereas the resident drinking water community remained similar between the loops (Figure 3). However, it is important to note that the bacterial indicators already faced difficulties in untreated tap water and that the indigenous drinking water community, possibly through competition for nutrients, inhibited the growth of the spiked bacterial indicators (Favere et al., 2021; van Bel et al., 2021; Van Nevel et al., 2013; Vital et al., 2012). Additionally, this invasion experiment was only performed once, repeating the experiment would have strengthened the findings.

### 4.4 Is membrane filtration the holy grail to produce biostable water?

In summary, while nutrient limitation is considered a key factor, UF and NF alone might not be the ultimate solution to prevent microbial regrowth and ensure biostability, as it was seen that the bulk cell concentrations remained unaffected. This study demonstrates that preventing regrowth requires more than nutrient limitation of the bulk water alone, as it could be that leaching from pipe material leaching and the use of non-sterile pipes and IBC’s potentially influences microbial growth as well. Membrane filtration is only one of many aspects and innovations, such as source protection, pretreatment, water reuse, smart storage solutions, biological online monitoring etc. that is, and in the future certainly will be, important to provide safe and qualitative drinking water. To produce microbiologically safe drinking water, safeguarding and monitoring the water sources, is as important, as implementing large-scale membrane filtration techniques poses practical challenges. Especially since these systems are energy-intensive, susceptible to membrane fouling, and require chemicals to clean (Jafari et al., 2021; Smeets et al., 2009; Van Der Bruggen et al., 2003). For a lot of existing drinking water production plants, there is no need to implement membranes in their production process to produce biostable water. However, the use of membranes could provide new opportunities for water reuse and the production of drinking water of alternative sources (e.g., brackish water, desalination), as it is also able to remove more than only bacteria and nutrients (i.e., organic micropollutants, antibiotics and antibiotic resistance genes) (Duarte et al., 2022).

## 5 Conclusion

Over the course of a 7-week experiment, NF-, UF-, and untreated tap water were added to a pilot-scale DWDS. This study demonstrated that membrane treated waters, particularly NF, reduced TOC and all MW fractions. Introduction of NF- and UF-treated water in DWDS left the mature biofilm unaffected regarding bacterial community composition and cell density. Biofilms that were only exposed to the treated waters, showed a lower cell densities when NF-treated water was recirculated, indicating that nutrient limitation can restrict biofilm development. Nonetheless, the biofilm community composition remained stable across treatments for the young biofilms, suggesting that colonization may be more influenced by existing biofilm communities and material-derived nutrients (e.g., from PVC piping) than by changes in the bulk water composition alone.

Microbial (re)growth was observed in all loops, potentially due to residual bacteria, and the use of non-sterile system components rather than biofilm detachment. Despite the treated waters being low in nutrients, increases in bulk cell concentrations reached a similar cell concentration after 7 days of recirculation across all loops, supporting the hypothesis that selection pressure under nutrient-limited conditions favors specific microbial populations with high substrate affinity.

An invasion experiment revealed that unwanted microorganisms (e.g., *Aeromonas media*, *Pseudomonas putida*, *Serratia fonticola*) exhibited a faster decay in NF- and UF-treated water compared to untreated tap water, further highlighting the potential of membrane filtration to enhance the biostability of drinking water. These findings reinforce the importance of nutrient limitation, particularly through removal of AOC and the high MW fraction as a tool for microbial growth control in DWDS. Our results provide new insights into how nutrient availability shapes microbial persistence in drinking water distribution systems.

## Data Availability

The original contributions presented in the study are publicly available. This data can be found in here: https://www.ncbi.nlm.nih.gov/, accession number PRJNA1298809.
