# Supplementary material for: Membrane filtration reduces nutrient availability and invasion potential in drinking water systems, without affecting mature biofilms
Source: Front Microbiol. 2025 Aug 13;16:1622038. doi: 10.3389/fmicb.2025.1622038 (PMC12380793; doi:10.3389/fmicb.2025.1622038)
Supplement: Supplementary file 1 [file Data_Sheet_1.docx]

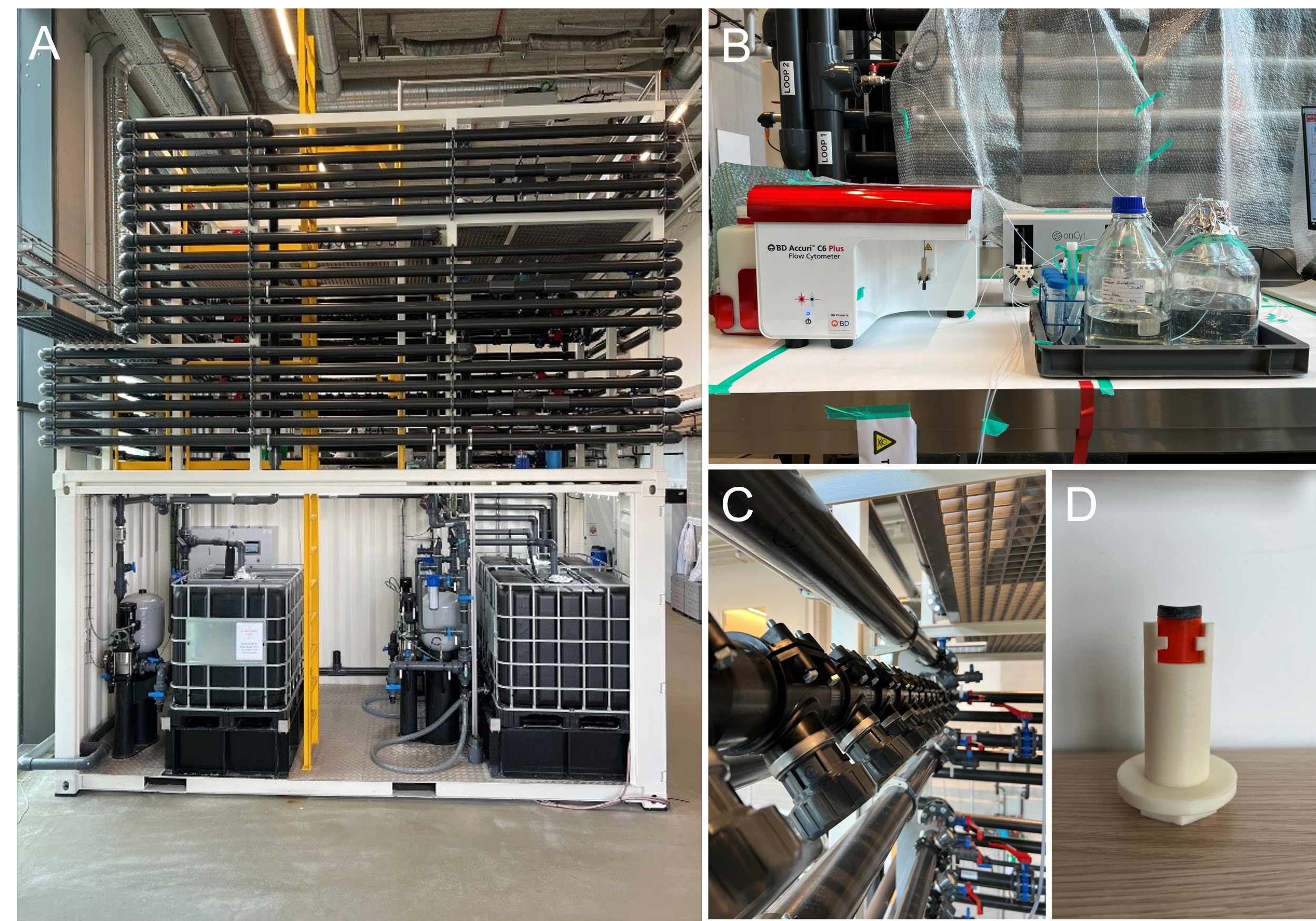


Figure S1: (A) The drinking water distribution pilot comprises three identical loops of 100 m each, connected to a non-translucent IBC. The structure measures 5.2 m × 2.6 m. (B) Implementation of online microbial monitoring: An Accuri™ C6 Plus flow cytometer (left) is coupled with an onCyt© autosampler, facilitating automated sampling from the pilot and cleaning solutions (right). (C) Biofilm sampling involves the use of coupons for undisruptive examination. These coupons are installed on a pipe using a system designed to resist pressure. (D) The coupon when removed from the pipe. It consists of a white holder (3.4 cm of diameter, 9.5 cm long), with a small insert (2.2 cm of diameter, 2.5 cm long) that can be placed within it. The top of this insert, which comes into contact with the water, is made of PCV-U, the same material as the pipes.


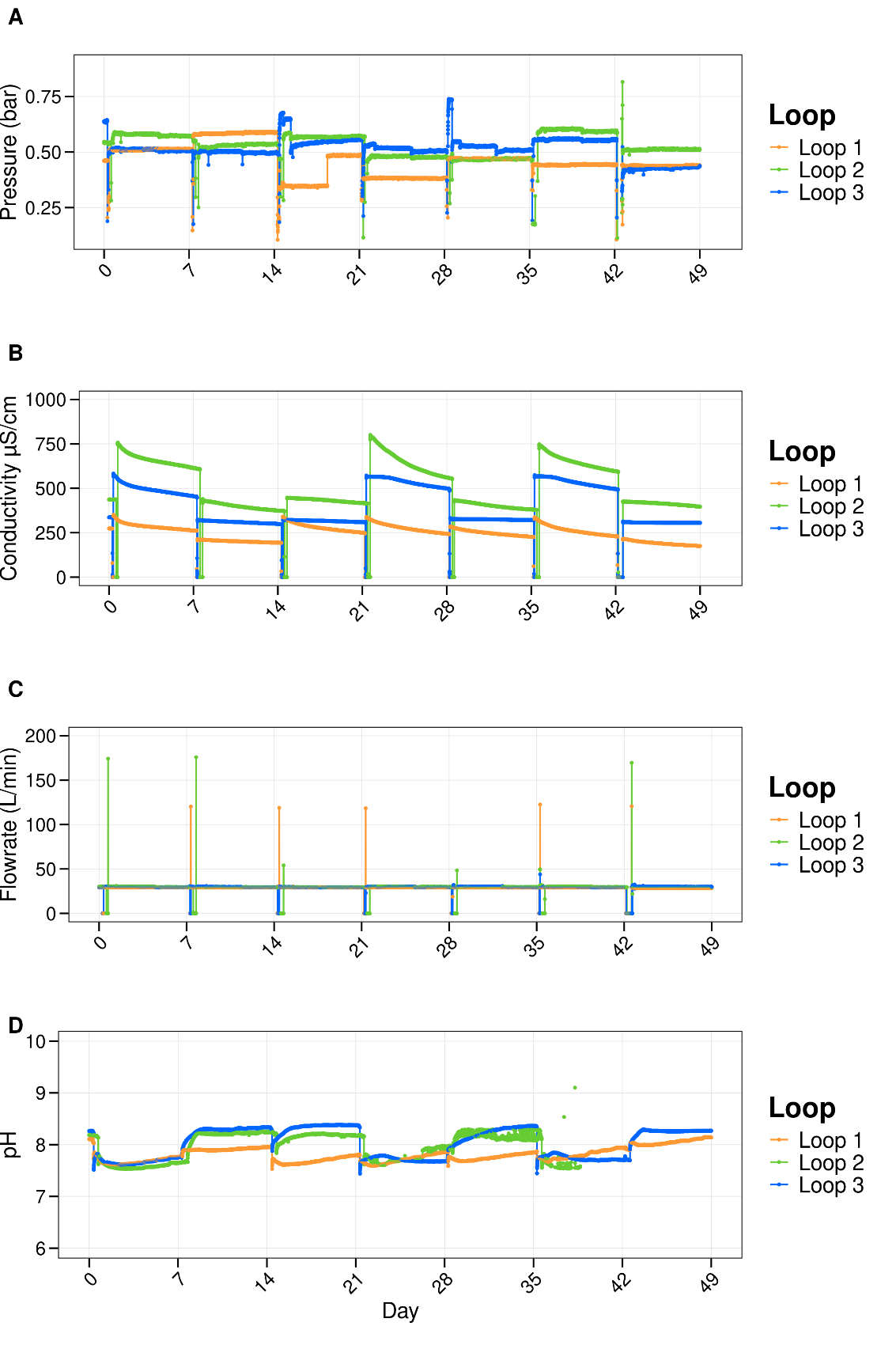


Figure S2: Overview of the physical parameters in the pilot distribution system measured by the online sensors over the entire 7-week experiment. (A) The pressure in bar (y-axis) in the pilot measured online over the course of the 7-week experiment. (B) The conductivity in µS/cm (y-axis) in the pilot measured online over the course of the 7-week experiment. (C) The flowrate in L/min (y-axis) measured online over the course of the 7-week experiment. (D) The pH (y-axis) measured online over the course of the 7-week experiment.


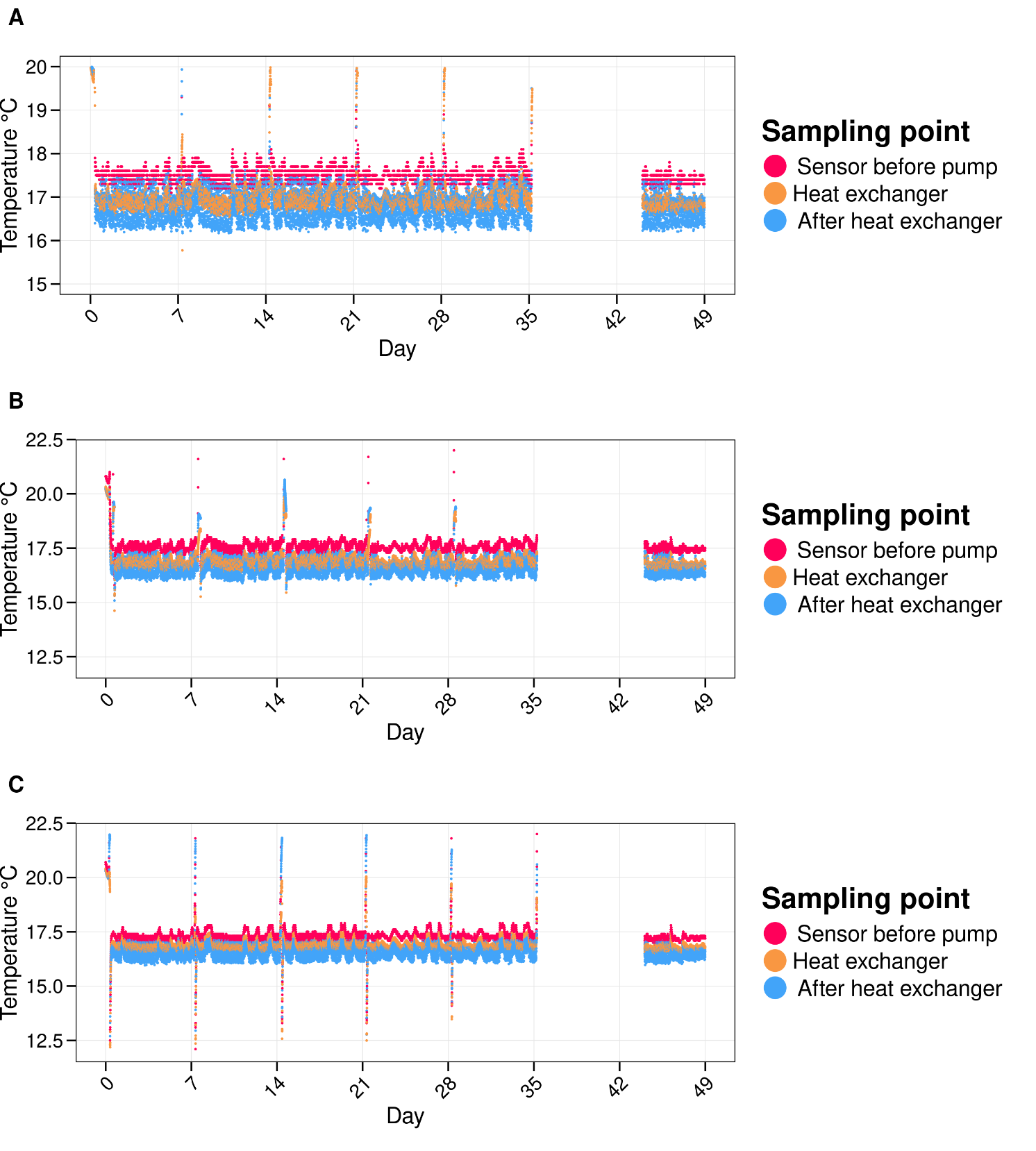


Figure S3: Overview of the temperature (°C) in the pilot distribution system measured by three online temperature sensors (placed before the pump, before the heat exchanger and after the heat exchanger) over the entire 7-week experiment for loop 1 recirculated with NF treated water (A), loop 2 recirculated with UF treated water (B) and loop 3 recirculated with untreated tap water (C). This demonstrated the continuous temperature maintained over the 7-week experiment.


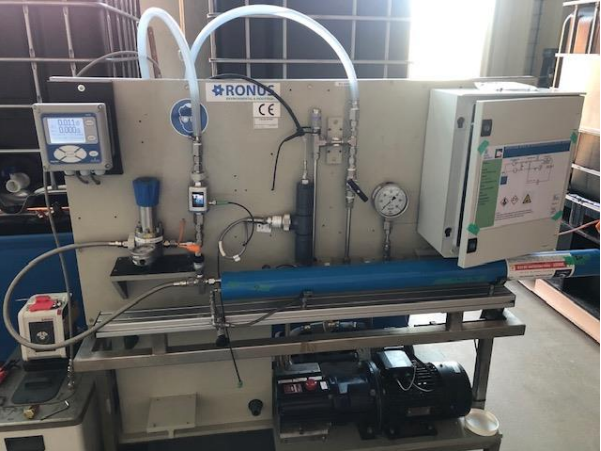


**Figure S4**: The membrane filtration unit used for the UF and NF treatment. Crossflow UF and NF was performed on this pilot-scale unit with a 2540-type spiral wound membrane element (2.5’’) with an active filtration area of 2.7 m². Feed and permeate tubing were connected to 1 m3 IBCs to ensure sufficient volume production.


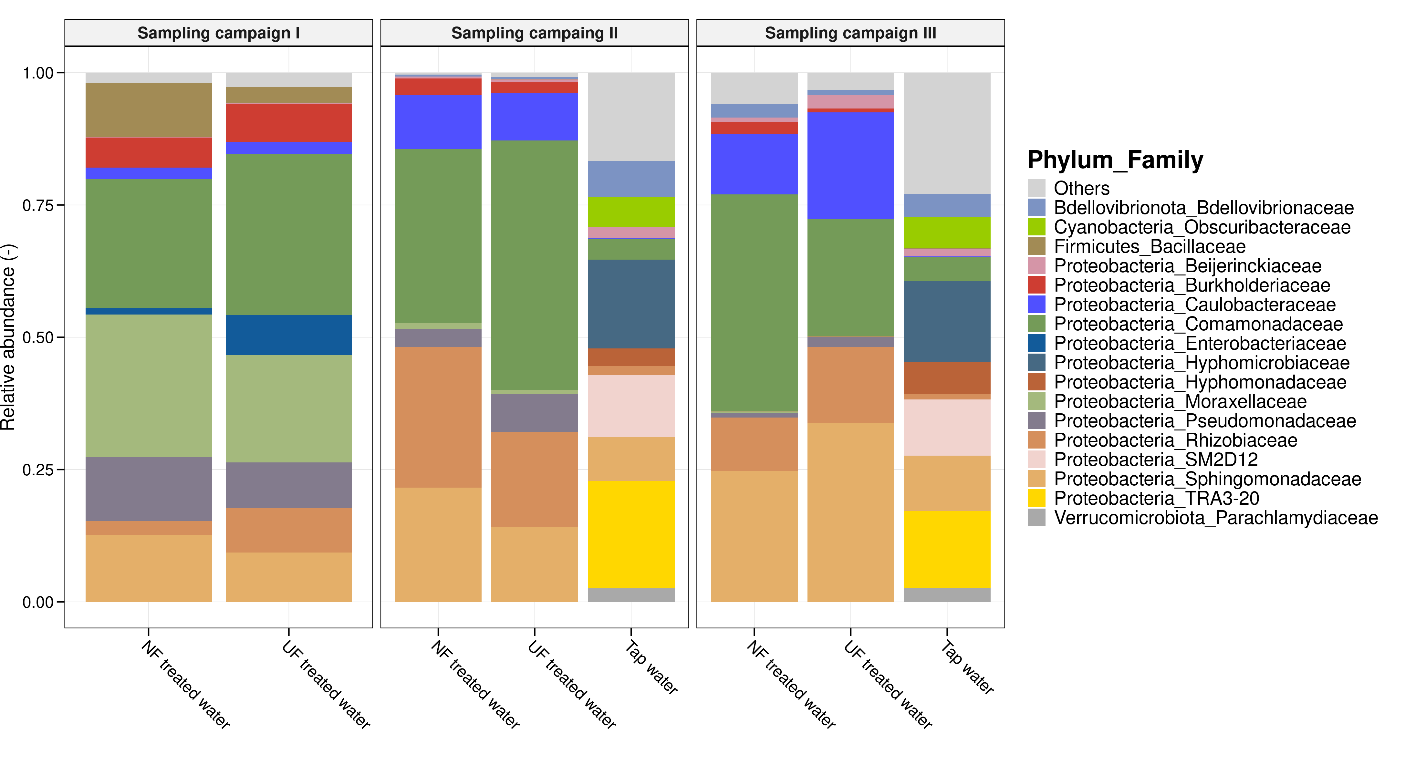


Figure S5: Relative abundances of the 17 most abundant families of treated water (NF and UF) and untreated water at the start of Experiment I, II, and III (n = 1 for each bar plot).


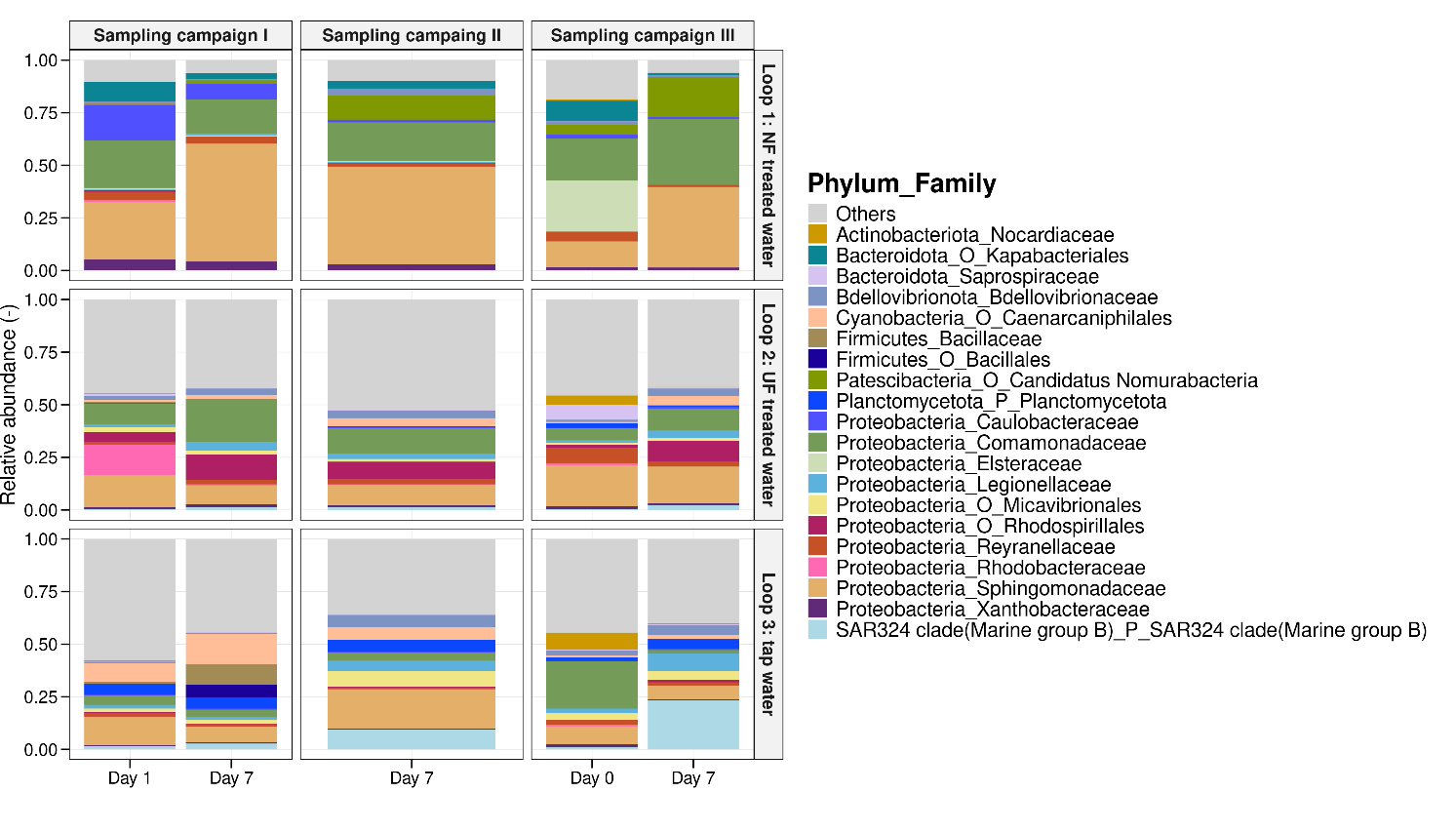


Figure S6: Relative abundances of the 20 most abundant families of the bulk water inside the DWDS pilot. At each timepoint, one water sample was taken per loop (n = 1).


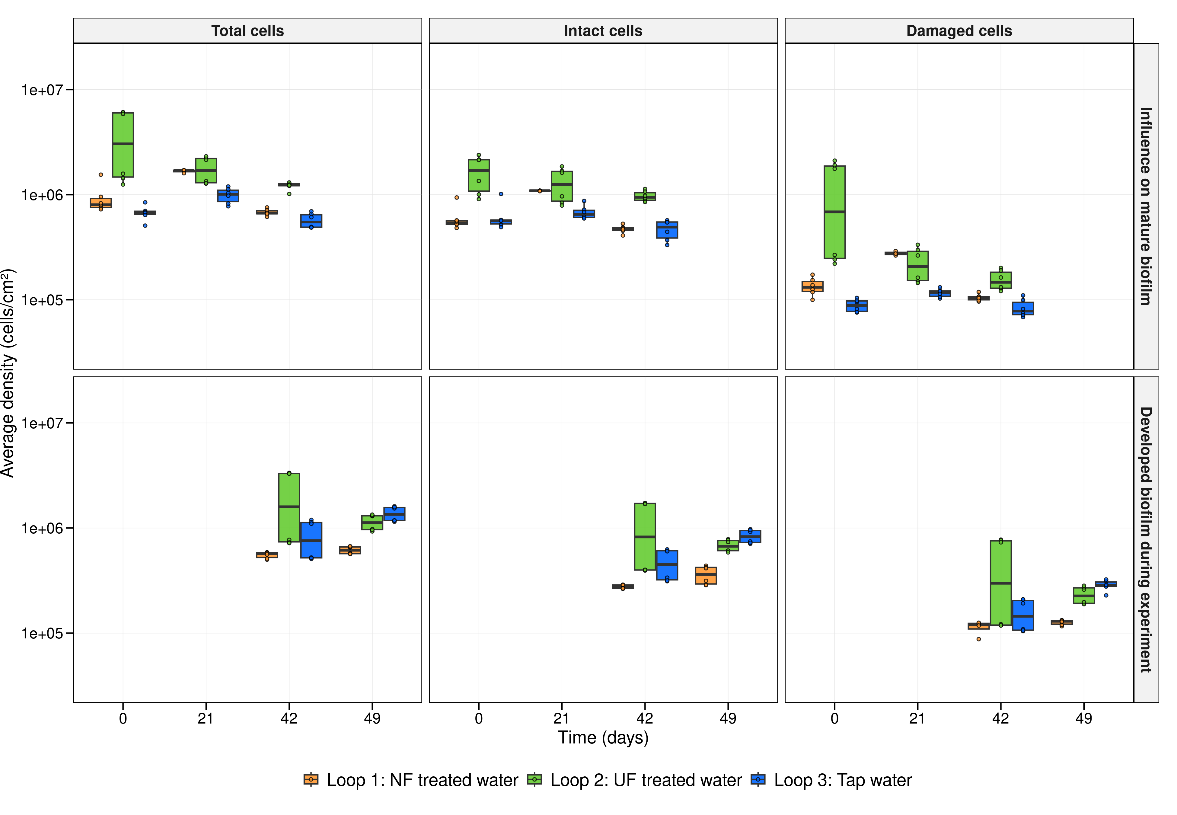


Figure S7: Average total, intact, and damaged cell density (cells/cm²) per timepoint of the microbial community in loop 1 (orange), loop 2 (green), and loop 3 (blue) for the mature biofilm and the biofilm that only received treated water.


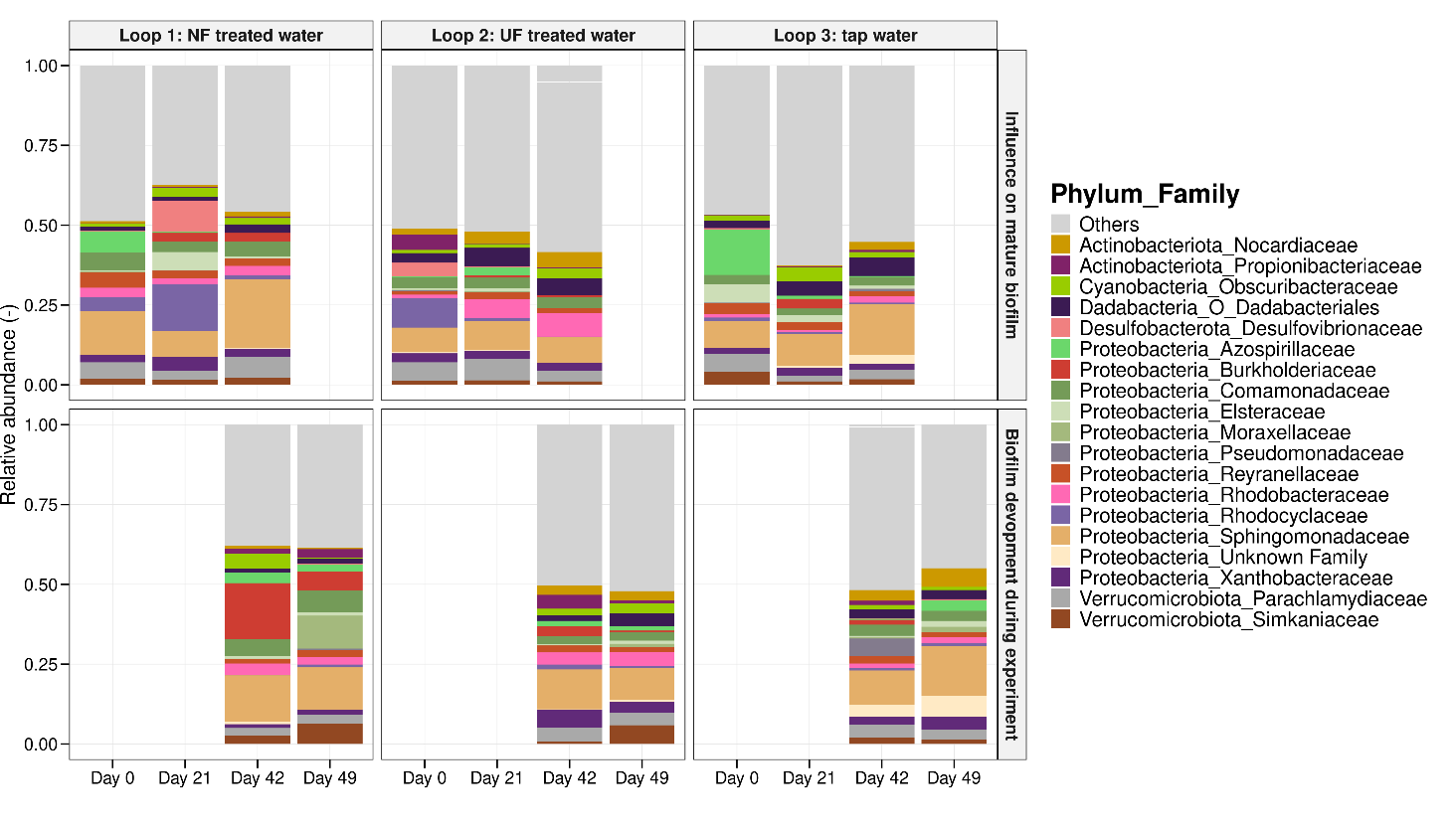


Figure S8: Relative abundances of the 19 most abundant families of the biofilm in each loop. Two biological replicates per timepoint were taken (n = 2).


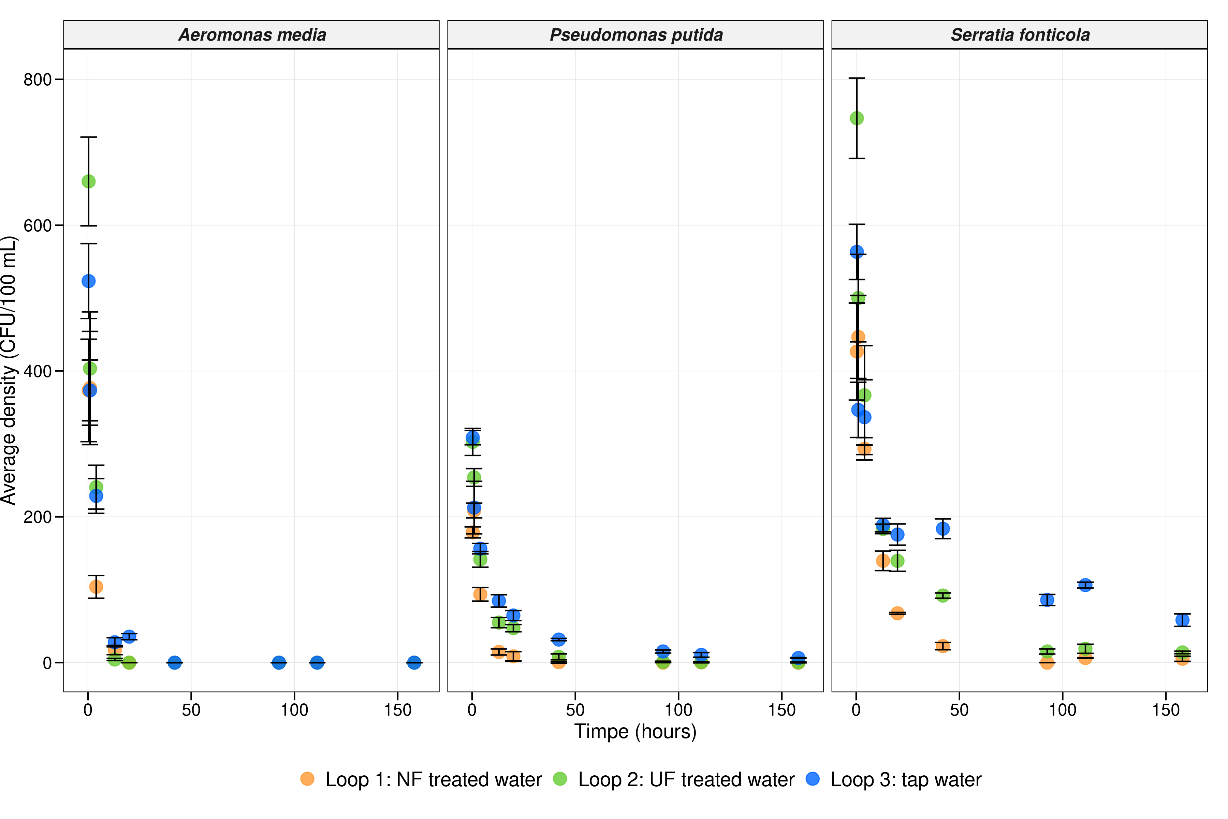


Figure S9: Average concentration (CFU/100 mL) in function of time (hours) for each invader in loop 1 (orange), loop 2 (green), and loop 3 (blue). Per timepoint, biological replicates (n = 3) were taken and corresponding error bars are shown in black.
